# Comparative Genome Analysis between *Agrostis stolonifera* and Members of the Pooideae Subfamily, including *Brachypodium distachyon*

**DOI:** 10.1371/journal.pone.0079425

**Published:** 2013-11-11

**Authors:** Loreto Araneda, Sung-Chur Sim, Jin-Joo Bae, Nanda Chakraborty, Joe Curley, Taehyun Chang, Maiko Inoue, Scott Warnke, Geunhwa Jung

**Affiliations:** 1 Stockbridge School of Agriculture, University of Massachusetts, Amherst, Massachusetts, United States of America; 2 Department of Bioresources Engineering, Sejong University, Seoul, Korea; 3 Department of Horticulture, University of Wisconsin, Madison, Wisconsin, United States of America; 4 Syngenta Seeds, Inc., Stanton, Minnesota, United States of America; 5 School of Ecology & Environmental System, Kyungpook National University, Sangju, Korea; 6 United States Department of Agriculure-Agricultural Research Service, Floral and Nursery Plants Research Unit, Beltsville, Maryland, United States of America; CNR, Italy

## Abstract

Creeping bentgrass (*Agrostis stolonifera*, allotetraploid 2n = 4x = 28) is one of the major cool-season turfgrasses. It is widely used on golf courses due to its tolerance to low mowing and aggressive growth habit. In this study, we investigated genome relationships of creeping bentgrass relative to the Triticeae (a consensus map of *Triticum aestivum*, *T. tauschii*, *Hordeum vulgare*, and *H. spontaneum*), oat, rice, and ryegrass maps using a common set of 229 EST-RFLP markers. The genome comparisons based on the RFLP markers revealed large-scale chromosomal rearrangements on different numbers of linkage groups (LGs) of creeping bentgrass relative to the Triticeae (3 LGs), oat (4 LGs), and rice (8 LGs). However, we detected no chromosomal rearrangement between creeping bentgrass and ryegrass, suggesting that these recently domesticated species might be closely related, despite their memberships to different Pooideae tribes. In addition, the genome of creeping bentgrass was compared with the complete genome sequence of *Brachypodium distachyon* in Pooideae subfamily using both sequences of the above-mentioned mapped EST-RFLP markers and sequences of 8,470 publicly available *A. stolonifera* ESTs (AgEST). We discovered large-scale chromosomal rearrangements on six LGs of creeping bentgrass relative to *B. distachyon*. Also, a total of 24 syntenic blocks based on 678 orthologus loci were identified between these two grass species. The EST orthologs can be utilized in further comparative mapping of Pooideae species. These results will be useful for genetic improvement of *Agrostis* species and will provide a better understanding of evolution within Pooideae species.

## Introduction

Angiosperm genomes vary in size and arrangement, even within closely related species. Despite frequent whole-genome duplications in angiosperms, the number of chromosomes tends to be less variable than genome size. Analyses of changes in genomic structure, such as specific gene rearrangements, insertions or deletions, provide an informative way to clarify relationships among lineages. In the grass family Poaceae, significant variation in DNA content and chromosome number suggests that genome evolution is fast and dynamic [1]. Poaceae is taxonomically classified into major subfamilies, Bambusoideae, Oryzoideae, Pooideae, Panicoideae, Arundinoideae, Chloridoideae, and Centothecoideae, which are further categorized into many tribes [2], [3]. Rice belongs to Oryzeae tribe under the Oryzoideae subfamily. The most important temperate forage and turfgrass species belong to the Pooideae subfamily, which is further classified into multiple major tribes, Poeae (*Poa* spp., *Lolium* spp., *Festuca* spp.), Triticeae (*Hordeum* spp., *Triticum* spp.), Agrostideae (*Agrostis* spp.), Aveneae (*Aveneae* spp.) and Brachypodieae (*Brachypodium* spp.).

Comparative genome studies in the grass family Poaceae are commonly carried out using rice as a model system due to its compact and well-annotated genome sequence. However, there are multiple complications that have been encountered in previous research; rapid change in the rice genome (due to segmental and tandem duplications, and gene movements) [4], lack of key biological features of a model organism (short plant stature and rapid life cycle) and the phylogenetic distance from the Pooideae subfamily including wheat, barley, and temperate forage and turfgrasses. All together these shortcomings of rice make it a poor model organism. Moreover, rice does not display all of the desirable traits applicable to study in temperate grasses, such as fungal resistance, freezing and heat tolerance, wear and injury tolerance, and others [5], [6].

For several reasons, *Brachypodium distachyon* has been proposed as an improved model system for temperate grasses over rice. *B. distachyon* is an annual wild grass member of the Brachypodieae tribe in the Pooideae subfamily. In addition to the small and completely sequenced genome, *B. distachyon* has other desirable biological features and a closer phylogenetic relationship to temperate cereals, forage and turfgrasses [5]–[9].

Comparative genome analysis using a common set of RFLP probes has demonstrated shared gene content and macrocollinearity of genes among related Poaceae species, such as maize, wheat, barley, oat and rice [10]–[13]. In underrepresented turfgrass species where whole-genome sequences or physical maps are not available, marker-based mapping [10]–[12], [14] and transcriptome-based genetic linkage map [15] are the best approaches for a better understanding of the whole-genome.

Species of the genus *Agrostis* are perennial, self-incompatible and C3 cool-season grasses native to the temperate and subarctic climates of Western Europe. Four main species are commonly accepted as turfgrass types: 1) creeping bentgrass *A. stolonifera* L. (2*n* = 4*x* = 28, A_2_ and A_3_ genomes), 2) velvet bentgrass *A. canina* L. (2*n* = 2*x* = 14, A_1_ genome), 3) colonial bentgrass *A. capillaris* L. (2*n* = 4*x* = 28, A_1_ and A_2_ genomes), and 4) redtop bentgrass *A. gigantea* L. (2*n* = 6*x* = 42, A_1_, A_2_, and A_3_ genomes). Creeping bentgrass is the most widely used for golf courses because of its fine texture, excellent tolerance to low mowing, strong stoloniferous growth habit and cold tolerance. The genus *Agrostis* has been placed in the subfamily Pooideae of the grass family Poaceae that includes rice (*Oryza sativa* L.), wheat (*Triticum aestivum* L.), barley (*Hordeum vulgare* L.), maize (*Zea mays* L.), oat (*Avena sativa* L.), ryegrass (*Lolium* spp.), sorghum (*Sorghum bicolor* L.), and *B. distachyon*. *Agrostis* has been assigned to the tribe Aveneae, which also includes oat [2], [13]. Furthermore, two tribes Aveneae and Poeae are closely related [16] and in some phylogenies have been combined [17]. However, Quintanar et al. [18] recently proposed that Poeae tribe could expand to include the former Aveneae, Poeae, and Seslerieae lineages and be split into multiple subtribes; Aveninae, Koelerinae, Agrostidinae, Loliinae, Poinae, and others according to phylogenetic structure of plastid and ITS sequences. The ITS analysis further suggested that the genus *Agrostis* belongs to the Agrostidinae and is para/polyphyletic.

A few studies using molecular marker-based linkage mapping and trait mapping have been conducted in *Agrostis* species. The first genetic linkage map of creeping bentgrass [19] was developed from a full-sib reference mapping population by crossing two naturalized clones (549 and 372) collected from old golf courses in Wisconsin [20]. The linkage map covered a distance of 1,110 cM, and contained 14 linkage groups, which were mapped with 424 loci, including expressed sequence tag-restriction fragment length polymorphism (EST-RFLP), randomly amplified polymorphic DNA (RAPD), and amplified fragment length polymorphism (AFLP). Seven pairs of the homoeologous linkage groups were identified based on duplicated RFLP loci and numbered according to syntenic chromosomes of ryegrass and the Triticeae [19]. In addition, the first genetic linkage map of colonial bentgrass using the backcross population of colonial bentgrass x creeping bentgrass (recurrent parent) mapped with AFLP and gene-based markers were published [21]. Recently, QTL mapping of field resistance to dollar spot caused by *Sclerotinia homoeocarpa* was investigated in an enhanced map of the 549×372 population using additional EST-RFLP loci [22].

Comparative genome relationships between *Agrostis* and other members of the Poaceae species need to be established so that genome resources from well-studied species can be utilized to understand the genomes of underrepresented species for improvement. One objective of this study was to investigate comparative genome relationships of creeping bentgrass relative to the Triticeae, ryegrass, oat, and rice using a common set of EST-RFLP markers previously mapped. A second objective of this study was to explore the utility of *B. distachyon* as a model system for other members of Pooideae subfamily. To do this, comparative genome analysis of creeping bentgrass was performed with the whole-genome sequences of *B. distachyon* using both publicly available sequences of 8,470 creeping bentgrass ESTs and sequences of the above-mentioned EST-RFLP markers.

## Results

### Creeping bentgrass linkage map based on EST-RFLP markers

**Figure 1:**
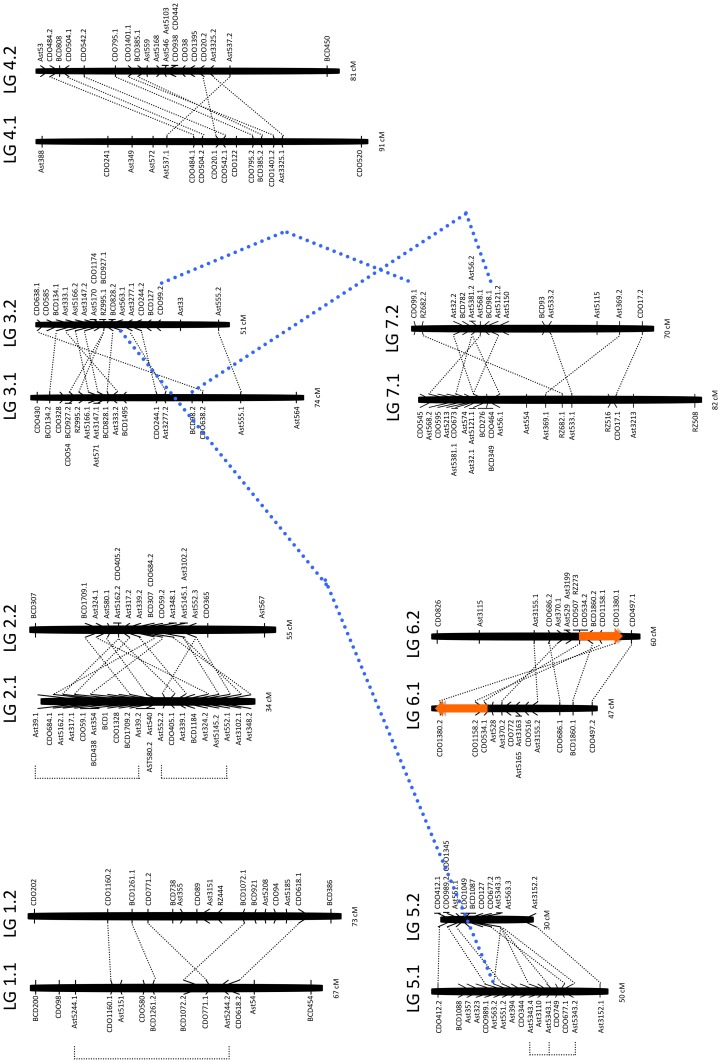
EST-RFLP genetic linkage map of creeping bentgrass. Two different creeping bentgrass diploid genomes are indicated by seven pairs of the homoeologous linkage groups (LGs) followed by “.1” or “.2”. The total map length in cM is shown on the bottom of each LG. The creeping bentgrass, barley, oat and rice cDNA probes used as RFLP markers are indicated as Ast, BCD, CDO and RZ, respectively followed by the probe number. The probe numbers plus ‘.1’, ‘.2’, ‘.3’ or ‘.4’ show duplicate loci detected by the same hybridization probe, which are connected by a dashed black line. Loci connected by a dashed bold blue line are detected between different LGs by the same hybridization probe. The segment on LGs 6.1 and 6.2, spanning three RFLP markers (CDO1380, CDO1158 and CDO534) superimposed by an orange arrow indicates an inversion and translocation between the two homoeologous LGs.

For comparative genome analysis in this study, we reconstructed a RFLP linkage map of creeping bentgrass using 229 EST-RFLP markers that were previously mapped to 14 linkage groups along with RAPD and AFLP markers [22]. The current map consisting of only RFLP markers provided similar marker order compared to the previous map but was 245 cM shorter in length. In brief, these RFLP markers were generated from 159 RFLP probes including 66 probes (42%) that generated more than 2 duplicated loci per probe (Table 1). A total number of 229 RFLP markers were separated into 14 linkage groups (LGs) at LOD thresholds ranging from 4.0 to 10.0, which were paired into 7 homoeologous LG sets (Figure 1). The numbering of each pair of the homoeologous LGs followed Chakraborty et al. [22]. A total of 865 cM was covered in the current linkage map and the genome coverage varied from 30 cM to 91 cM for each LG. The number of marker per LG ranged from 11 to 22 and an average interval between markers was 3.8 cM in size (Figure 1). Seven probes including Ast5244, Ast39, Ast5343, Ast552, Ast563, BCD98, and CDO99 generated markers that were duplicated within and/or between LGs. The distal end segments of two homoeologous LGs 6.1 and 6.2 showed an evidence of inversion and translocation (Figure 1). The sequences of the 49 bentgrass Ast probes mapped in the current study were BLAST searched, and 39 of them showed similarity to known genes. Moreover 47 and 32 of the Ast probes showed their putative chromosomal locations in rice and wheat, respectively (Table S1).

**Table 1:** Duplicate RFLP loci mapped in the 549×372 mapping population of creeping bentgrass.

Source of probe a	No. of probes generating multiple loci				
	1 locus	2 loci	3 loci	4 loci	Total
Ast	37	28	2	1	68
BCD	20	9	0	0	29
CDO	32	24	0	0	56
RZ	4	2	0	0	6
Total	93	63	2	1	159

Duplicate RFLP loci derived from 159 heterologous cereal and creeping bentgrass EST-RFLP probes that were mapped in the 549×372 mapping population of creeping bentgrass.

a Ast, BCD, CDO, and RZ probes derived from creeping bentgrass, barley, oat, and rice cDNAs, respectively.

### Genome relationships of creeping bentgrass relative to rice, the Triticeae, oat, and ryegrass *Rice*

**Figure 2:**
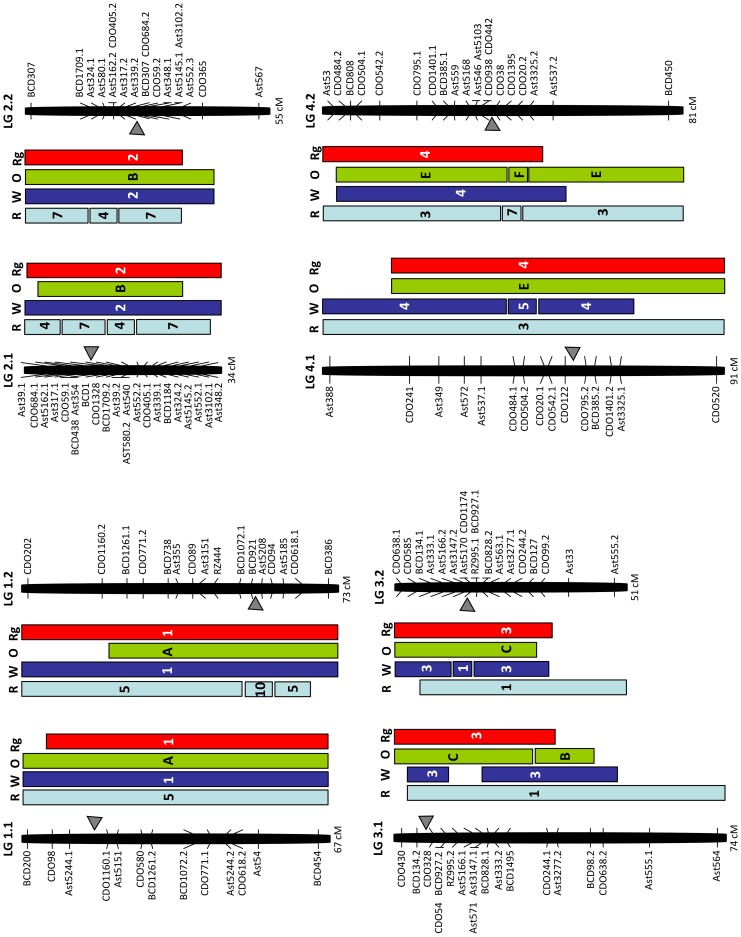
Comparative genome relationships on creeping bentgrass linkage groups 1–4 relative to rice, the Triticeae, oat, and ryegrass. Comparative genome relationships between creeping bentgrass genetic linkage map and the genetic maps of rice (R), the Triticeae (W), oat (O), and ryegrass (Rg), respectively, represented by a colored box. The markers shown on the right or left side of each linkage group correspond to those mapped in the creeping bentgrass linkage map shown in Figure 1. The number or letter inside the color boxes represents the segments of chromosomes or linkage groups from each of the genomes (R, W, O, Rg) that are syntenic to the bentgrass linkage groups. The arrowheads indicate the deduced location of the centromere in bentgrass from the comparisons with Triticeae chromosomes. The total map distances (cM) are shown on the bottom of each linkage group.

**Figure 3:**
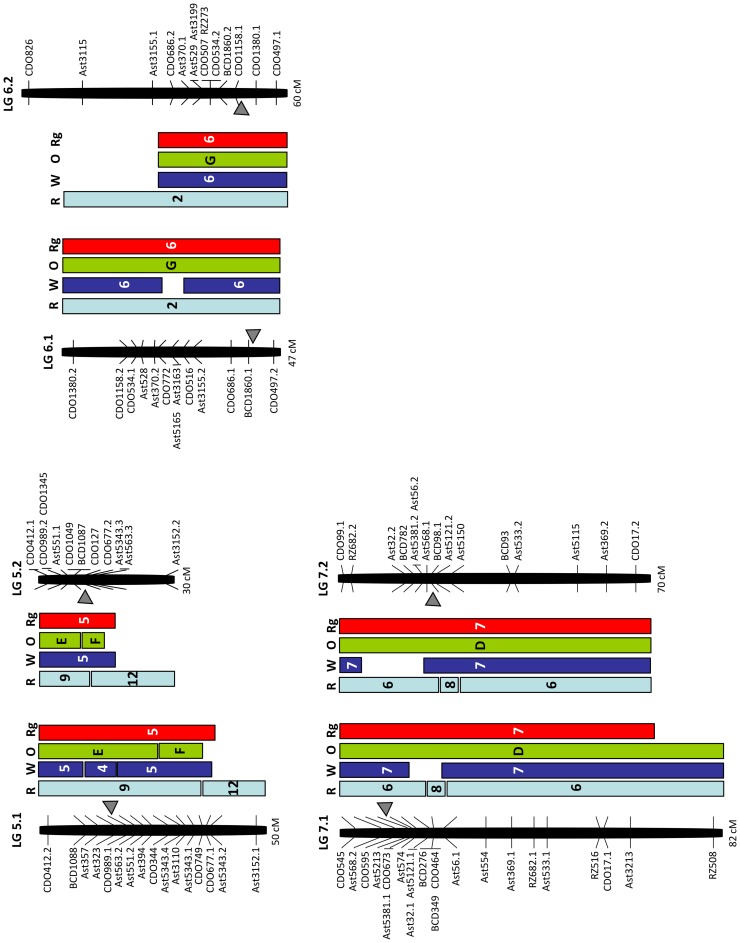
Comparative genome relationships on creeping bentgrass linkage groups 5–7 relative to rice, the Triticeae, oat, and ryegrass. Comparative genome relationships between creeping bentgrass genetic linkage map and the genetic maps of rice (R), the Triticeae (W), oat (O), and ryegrass (Rg), respectively, represented by a colored box. The markers shown on the right or left side of each linkage group correspond to those mapped in the creeping bentgrass linkage map shown in Figure 1. The number or letter inside the color boxes represents the segments of chromosomes or linkage groups from each of the genomes (R, W, O, Rg) that are syntenic to the bentgrass linkage groups. The arrowheads indicate the deduced location of the centromere in bentgrass from the comparisons with Triticeae chromosomes. The total map distances (cM) are shown on the bottom of each linkage group.

Eighty-three common loci on the rice and bentgrass genetic maps covered 82% of the bentgrass map (Table 2). Twenty-nine segments derived from all of the rice chromosomes (except for chromosome 11) cover all bentgrass LGs (Figures 2 and 3). Rice chromosome 11 was represented only by BCD808 on LG4.2 and by three duplicated loci generated from Ast563 probe, which were mapped on LGs 5.1, 5.2, and 3.2. With the exception of a few non-syntenic loci, both homoeologous pairs of LGs 3 and 6 showed the most conserved syntenic relationships with the homologous chromosomes 1 and 2 of rice, respectively. The other five bentgrass LGs (1, 2, 4, 5, and 7) showed large-scale chromosomal rearrangements relative to rice. Bentgrass LG1.2 was comprised of two syntenic segments from rice chromosomes 5 and 10. A segment containing BCD921 and CDO94 from rice chromosome 10 was placed between two segments of rice chromosome 5. Only CDO98 on LG1.1 represents rice chromosome 10. Bentgrass LGs 2.1 and 2.2 are represented by segments of rice chromosomes 4 and 7. Bentgrass LG4.2 is represented by the insertion of rice chromosome 7 segment between two distinct segments of rice chromosome 3. Bentgrass LGs 5.1 and 5.2 are represented by two segments of rice chromosomes 9 and 12. Lastly, LGs 7.1 and 7.2 are represented by the insertion of a segment of rice chromosome 8 between two distinct segments of rice chromosome 6.

**Table 2:** Genome comparison between creeping bentgrass and the Triticeae, oat, ryegrass and rice based on common heterologous and homologous RFLP markers.

Species	Number of common loci	Number of conserved syntenic blocks	Number of chromosomal rearrangements	Genome coverage (%) a
*Triticum* b	53	24	3	98
*Lolium* c	104	14	0	92
*Avena* d	42	19	4	76
*Oryza sativa* e	83	29	8	82

Genome comparisons of the 549×372 map of *Agrostis stolonifera* with previously published maps of *Triticum*, *Lolium*, *Avena*, and *Oryza sativa* based on a common set of heterologous cereal and creeping bentgrass cDNA probes.

a Genome coverage in bentgrass (%)  =  (x/y) x 100, where x  =  total map length (cM) represented in bentgrass by probes mapped in each species (Triticeae, ryegrass, oat, or rice) and y  =  total map length (cM) of bentgrass.

b *Triticum* map was derived from the consensus map for *T. aestivum*, *T. tauschii*, *H. vulgare*, and *H. spontaneum* [44].

c *Lolium* map was derived from diploid MFA x MFB interspecific cross between *L. multiflorum* Lam. and *L. perenne* L. [30].

d *Avena* map was derived from diploid interspecific cross between *A. atlantica* and *A. hirtula* [12].

e *Oryza sativa* map was derived from Gramene (http://www.gramene.org/cmap) and published map of Ahn and Tanksley [45].

**The Triticeae.** The 53 EST-RFLP loci with known map locations in the Triticeae were evenly distributed on seven pairs of creeping bentgrass LGs (Figures 2 and 3). These markers covered 98% of the bentgrass map (Table 2). Bentgrass LGs 1, 2, 6, and 7 showed a high level of synteny with corresponding homologous chromosomes of the Triticeae. A chromosomal rearrangement on bentgrass LG3.2 relative to the Triticeae chromosomes 1 and 3 was detected. Bentgrass LG3.2 is represented by the insertion of a segment (covered by Ast5166.2 and Ast5170) of the Triticeae chromosome 1 between two distinct segments of the Triticeae chromosome 3. Bentgrass LGs 4.1 and 4.2 contained large-scale chromosomal rearrangements relative to the Triticeae chromosomes 4 and 5. A distal end segment of the Triticeae chromosome 5 flanked by CDO484.1 and CDO504.2 loci on LG4.1 was inserted into the Triticeae chromosome 4. For LG4.2 the same segment, flanked by the duplicated loci CDO484.2 and CDO504.1, was observed but due to disruption by a non-syntenic marker (BCD808), a block of synteny could not be established. In addition, bentgrass LG5.1 was represented by segments of the Triticeae chromosomes 4 and 5. A segment of the Triticeae chromosome 4 flanked by Ast357 and CDO989.1 loci was inserted into the Triticeae chromosome 5. Results indicated that bentgrass LGs 4.1 and 5.1 consisted of reciprocal translocation of the Triticeae chromosomes 4 and 5 (Figures 2 and 3). In total, we detected three large-scale chromosomal rearrangements in LGs 3, 4, and 5, which can differentiate the bentgrass genome from the Triticeae genome.

**Oat.** Relatively low coverage of the bentgrass map (76%) was detected by 42 common loci with oat and resulted in a weak syntenic block on bentgrass LG2 (Table 2). Our comparative map indicated that bentgrass LGs 4 and 5 consisted of rearrangements (reciprocal translocations) of oat chromosomes E and F (Figures 2 and 3). Bentgrass LG4.2 was composed of two segments of oat chromosomes E and F. A segment from the most distal area in oat chromosome F, between CDO1395 and CDO20.2 loci, was inserted between CDO38 and BCD450 loci in oat chromosome E. Bentgrass LGs 5.1 and 5.2 also included two segments of chromosomes E and F. Therefore, bentgrass LGs 4 and 5 were differentiated from oat chromosomes E and F. In addition, a large-scale chromosomal rearrangement on bentgrass LG3.1 relative to oat chromosomes B and C was detected. The LG3.1 is represented by attachment of a segment (flanked by CDO244.1 and BCD98.2) of the oat chromosome B to the distal end of oat chromosome C.

**Ryegrass.** One hundred-four common loci were assigned to their locations on the ryegrass map and covered 92% of the bentgrass map (Table 2). Except for a low number of non-syntenic loci, all seven bentgrass LGs have complete syntenic relationships with those of ryegrass (Figures 2 and 3). Even though bentgrass LGs 4 and 5 showed chromosomal rearrangements relative to the Triticeae and oat, no evidence of large-scale chromosomal rearrangement at the present map resolution was observed between bentgrass and ryegrass.

### Comparative genome analysis between creeping bentgrass and *B. distachyon*

**Figure 4:**
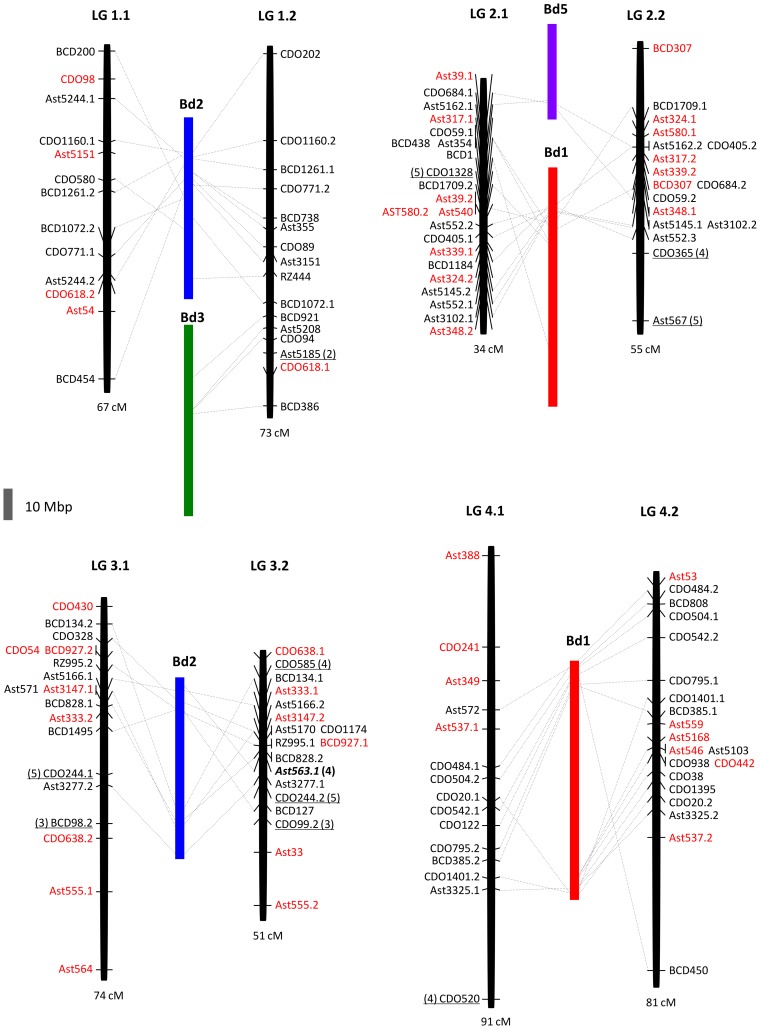
Comparative genome relationship on creeping bentgrass linkage groups 1–4 relative to *Brachypodium distachyon*. Comparative genome relationship between creeping bentgrass genetic linkage map and chromosomes of *B. distachyon* by determining the chromosomal location of sequences of the EST-RFLP markers mapped on the creeping bentgrass linkage map. The black bar represents each of the bentgrass linkage groups (total length in cM, below the bar) as shown in Figure 1. The colored bars represent each of *B. distachyon* chromosomes (Bd). The collinearity is represented by a dashed black line that links the RFLP markers with the highly similar sequences located in *B. distachyon* chromosomes. Markers in red have no significant sequences similarity with *B. distachyon* genome. Underlined markers have significant sequences similarity with *B. distachyon* chromosomes indicated in parenthesis. Markers in bold and italics are duplicated between *B. distachyon* chromosomes indicated in parenthesis. The grey scale bar on the left bottom of the Figure is 10 Mbp of *B. distachyon* genome.

**Figure 5:**
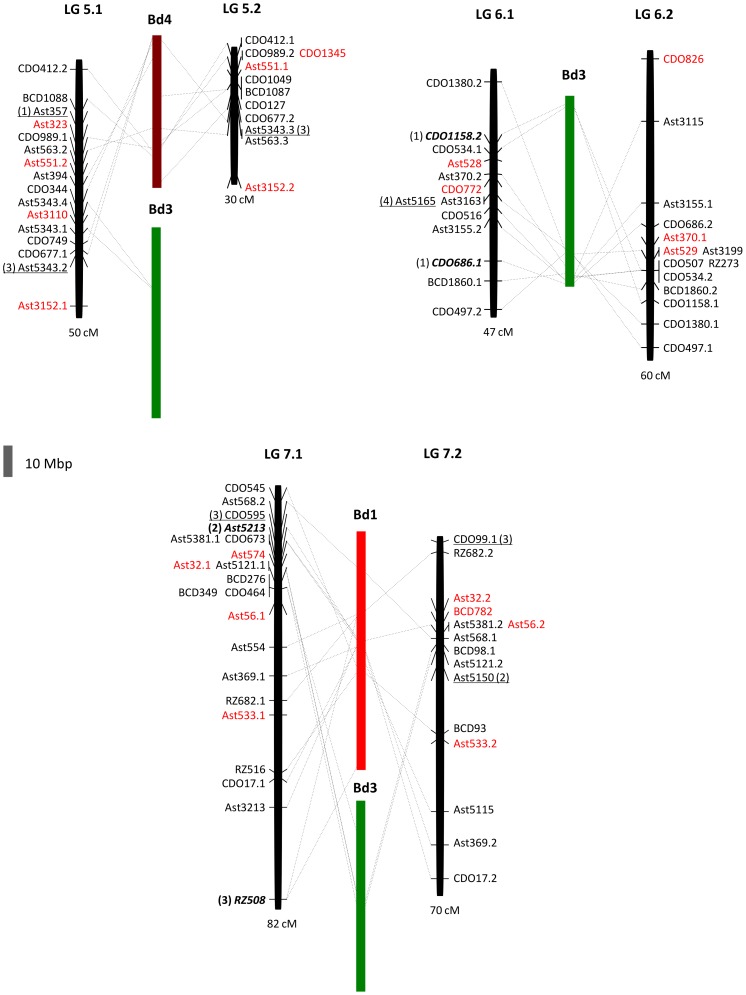
Comparative genome relationship on creeping bentgrass linkage groups 5–7 relative to *Brachypodium distachyon*. Comparative genome relationship between creeping bentgrass genetic linkage map and chromosomes of *B. distachyon* by determining the chromosomal location of sequences of the EST-RFLP markers mapped on the creeping bentgrass linkage map. The black bar represents each of the bentgrass linkage groups (total length in cM, below the bar) as shown in Figure 1. The colored bars represent each of *B. distachyon* chromosomes (Bd). The collinearity is represented by a dashed black line that links the RFLP markers with the highly similar sequences located in *B. distachyon* chromosomes. Markers in red have no significant sequences similarity with *B. distachyon* genome. Underlined markers have significant sequences similarity with *B. distachyon* chromosomes indicated in parenthesis. Markers in bold and italics are duplicated between *B. distachyon* chromosomes indicated in parenthesis. The grey scale bar on the left bottom of the Figure is 10 Mbp of *B. distachyon* genome.

The comparative analysis between creeping bentgrass and *B. distachyon* was conducted using BLASTN to anchor physical positions of the mapped EST-RFLP marker sequences in the genome of sequenced *B. distachyon* (Figures 4 and 5, Table S2). One hundred fourteen out of the 160 EST-RFLP markers with significant alignments (E ≤ 1×10^−10^) to *B. distachyon* genome were used for analysis of the comparative relationship between these two species. Twenty-four segments derived from all five *B. distachyon* chromosomes were distributed in all 14 bentgrass LGs (Figures 4 and 5). A block of synteny with chromosome 5 of *B. distachyon* was only established at the two markers CDO684 and Ast5162 duplicated on LGs 2.1 and 2.2. Markers CDO1328 on LG2.1, Ast567 on LG2.2, CDO244.1 on LG3.1 and CDO244.2 on LG3.2 also represent chromosome 5 of *B. distachyon*. The most conserved syntenic relationships were found between LG1.1 and chromosome 2, LGs 3.1–3.2 and chromosome 2, LGs 4.1–4.2 and chromosome 1, LG5.2 and chromosome 4, and LGs 6.1–6.2 and chromosome 3 of *B. distachyon*. Out of them, the largest inferred region showing 14 syntenic loci were detected between bentgrass LG4.2 and *B. distachyon* chromosome 1. However, on LGs 3.1 and 3.2, there still remain large regions with unknown syntenic relationship to sections of *B. distachyon* chromosomes.

The rest of the bentgrass LGs showed large-scale chromosomal rearrangements (LGs 1.2, 2.1–2.2, 5.1, and 7.1–7.2). Bentgrass LG1.2 presents synteny with two segments of *B. distachyon* chromosomes 2 and 3 and LGs 2.1and 2.2 have two blocks of synteny with chromosomes 1 and 5 (Figure 4). Linkage group 5.1 is represented by the insertion of a segment of chromosome 3 between two segments of chromosome 4. A similar rearrangement exists in LGs 7.1 and 7.2 but in these cases the segment of chromosome 3 is placed between two segments of *B. distachyon* chromosome 1 (Figure 5).

**Figure 6:**
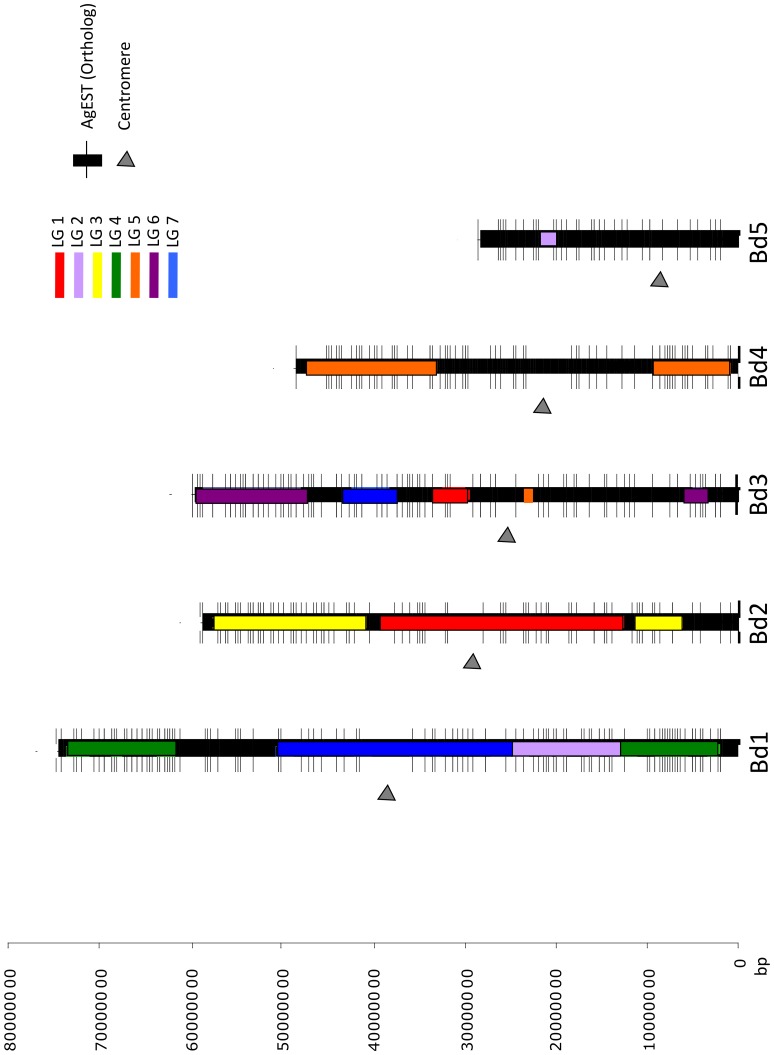
Creeping bentgrass ESTs orthologous to *Brachypodium distachyon*. Creeping bentgrass ESTs (AgEST) orthologous to *B. distachyon* genome and their location in the physical map of *B. distachyon* (in base pairs). Bd1, Bd2, Bd3, Bd4 and Bd5 represent the *B. distachyon* chromosomes (1 to 5) and horizontal lines denote the position of the 678 AgEST orthologs. The segments of a seven-color code (LGs 1–7) indicate the creeping bentgrass linkage groups that have synteny with a specific region of *B. distachyon* chromosomes. The arrowheads indicate the centromeric region.

The orthology analysis between 8,470 *A. stolonifera* ESTs (AgEST) and *B. distachyon* genome showed 678 non-redundant AgEST orthologs to *B. distachyon*: 196 AgEST in Bd ch1 (29%), 136 AgEST in Bd ch2 (20%), 151 AgEST in Bd ch3 (22%), 104 AgEST in Bd ch4 (15%), and 91 AgEST in Bd ch5 (14%) (Figure 6). Ninety-five percent of these orthologous loci corresponded to unique loci and the rest were found duplicated among and within *B. distachyon* chromosomes (Table S3).

## Discussion

*Agrostis* species are suggested to have the most complex genome structures among other genera within the grass family [23]. Previous cytological studies [24]–[26], disomic inheritance of polymorphic isozymes [27], RFLP-based linkage map with duplicated markers likely originating from the two subgenomes (A_2_ and A_3_) and the ratio of coupling vs. repulsion linkages between markers [19] suggest that creeping bentgrass (*A. stolonifera*) is a strict allotetraploid comprised of two A_2_A_2_ and A_3_A_3_ subgenomes. At the current map resolution, a high percentage (42%) of the mapped RFLP markers were duplicated and evenly distributed on the seven pairs of homoeologous LGs (Figure 1), which further confirm an allotetraploid origin [19], [24]–[27]. Recently cytogenetic analyses using fluorescence *in situ* hybridization (FISH) and genomic *in situ* hybridization (GISH) techniques revealed that intergenomic translocations and genome rearrangements might occur between the A_2_A_2_ and A_3_A_3_ subgenomes in creeping bentgrass [28]. These results further supported the strict allotetraploid nature of the creeping bentgrass due to the nonrandom separation of the two subgenomes.

Evidence of inversion and translocation between distal end segments of the two homoeologous LGs 6.1 and 6.2 indicated that the two subgenomes of creeping bentgrass could have gone through major chromosomal rearrangements. However, further research is required to confirm whether these chromosomal rearrangements occurred before homoeologous chromosomes’ differentiation or after. Unfortunately, diploid bentgrass species with either A_2_ or A_3_ genome are presently unconfirmed, but recent gene sequences-based phylogenic analysis suggests that the genome designation for diploid velvet bentgrass should be the A_2_ genome [29]. Based on cytological evidence, colonial (*A. capillaris*) and velvet bentgrasses (*A. canina*) have been proposed to contain the genome compositions A_1_A_1_A_2_A_2_ and A_1_A_1_, respectively. Comparison of these two species’ maps with the same set of RFLP probes uniquely present in each genome may reveal the chromosomes of the A_2_ genome common in creeping, colonial, and velvet bentgrasses.

A high level of conserved synteny between creeping bentgrass and other grass species such as the Triticeae, ryegrass, oat and rice was observed in the current study. However, comparative mapping studies within Poaceae species revealed evidence of unique large-scale chromosomal rearrangements, even though the number of common RFLP markers utilized was limited. Kellogg [16] studied genome structure within the grass family using comparative mapping and suggested that all species belonging to the Pooideae subfamily have two unique chromosomal rearrangements (5-10-5 and 6-8-6) relative to rice. As a member of the Pooideae subfamily, creeping bentgrass in the current study clearly shows the 5-10-5 and the 6-8-6 rice chromosomal rearrangements on LGs 1 and 7, respectively as observed in ryegrass [30].

When we compare creeping bentgrass with rice at this level of map resolution, only four RFLP probes (CDO365, CDO520, BCD808 and Ast563) represent rice chromosome 11 in six different bentgrass LGs (2.2, 3.2, 4.1–4.2, and 5.1–5.2). The poor synteny with the rice chromosome 11 may be attributable to the high fraction of repetitive sequences previously described in rice chromosomes 11 and 12 (29.5% and 31.6%, respectively) producing a disruption of synteny [31]. This disruption of synteny could be resolved in the future with the use of an increased number of RFLP markers. However, the rest of the rice chromosomes were represented by syntenic regions of all seven creeping bentgrass LGs. Syntenic relationships between rice and creeping bentgrass using our creeping bentgrass RFLP-based linkage map were similar to the ones between rice and perennial ryegrass using a transcriptome-based genetic linkage map of perennial ryegrass [15].

Based on comparisons of morphological characters, bentgrass and oat belong to the same Aveneae tribe, while ryegrass belongs to the Poeae tribe [2]. However, results of our comparative genome analyses show a strong syntenic relationship between bentgrass and ryegrass, with only four non-syntenic markers. Furthermore, events of large-scale chromosomal rearrangements relative to oat were detected on bentgrass LGs 3, 4 and 5. These results suggest that bentgrass might be more closely related to ryegrass than to oat in terms of genome structure, despite a closer taxonomic affinity between bentgrass and oat. These results, combined with previous reports in the recently domesticated species ryegrass, might provide evidence to dispute the currently accepted taxonomic relationship among species within Pooideae subfamily, which Jones et al. [32] and Sim et al. [30] first brought into question. Additionally, Rotter et al. [33] carried out the first and only phylogenetic analysis that included two members of *Agrostis* genus (*A. stolonifera* “creeping bentgrass” and *A. capillaris* “colonial bentgrass”) based on sequences of conserved ortholog sets (COS). Results suggested that creeping and colonial bentgrasses were clustered more closely to tall fescue (*Festuca arundinacea*, tribe Poeae) than to oat (tribe Aveneae). Therefore, these recently domesticated species, creeping bentgrass and perennial ryegrass, might be closely related to each other and be “wild” grass species of the Poaceae family. However, macrocolinearity relationships between the creeping bentgrass and perennial ryegrass genomes can be underestimated since comparative mapping using low-copy cDNA as hybridization probes often identified paralogous rather than orthologous sequences [34]. Future studies should include the construction of a different creeping bentgrass genetic map based on EST-SSR markers with known positions in the recently published ryegrass linkage map to further investigate the genetic relationship between these two species [35], [36].

We detected a total of six chromosomal rearrangements between creeping bentgrass and *B. distachyon* but there was no evidence of chromosomal rearrangement on LGs 3.1–3.2, 4.1–4.2 and 6.1–6.2 (Figures 4 and 5). Nevertheless, we observed many changes in the order of markers within syntenic blocks across the different LGs, a situation that could indicate that gene movements within chromosomes have taken place with a relatively high frequency [37]. Despite representation of all *B. distachyon* chromosomes across all creeping bentgrass LGs, chromosome 5 of *B. distachyon* is significantly underrepresented (only two syntenic blocks defined by two markers on LGs 2.1–2.2, Figure 4). According to Vogel et al. [9] the high long terminal repeat (LTR) retrotransposon density found in this *B. distachyon* chromosome could cause syntenic disruptions. One method to establish a more accurate syntenic relationship with *B. distachyon* chromosomes would be to develop molecular markers from the creeping bentgrass ESTs orthologous to the *B. distachyon* genome identified in this study for a more targeted comparative genome analysis. As a preliminary attempt we have designed conserved intron-spanning primers (data not shown) based on *B. distachyon* genome sequences and creeping bentgrass EST orthologs [38]. These primers were screened in nine species from three tribes Aveneae, Poeae and Brachypodieae and around 70% of them amplified strong polymorphic bands in all the species. Conserved intron-spanning markers make them ideal for future linkage disequilibrium studies due to the features of the close proximity of introns to exons and the conserved position of introns.

A previous study detected the most robust QTL for dollar spot resistance on LG7.1 of the creeping bentgrass linkage map [22]. The same authors mentioned that the marker associated with QTL for sheath blight resistance in rice caused by *Rhizoctonia solani* is present close to the dollar spot resistance QTL on the bentgrass LG7.1. In addition, previous studies have located QTL for resistance to crown rust (*Puccinia coronata* f. sp. *lolii*) and powdery mildew (*Erysiphe graminis*) in linkage group 7 of the ryegrass genetic map [39], [40]. This information suggests that LG7.1 of creeping bentgrass may play a more important role in disease resistance than all other linkage groups for focus in future research.

When we examined the putative function of the complete data set of creeping bentgrass ESTs used in this study, 13 of the ESTs showed similarity to genes associated with host defense or disease resistance including leucine rich repeat and protein kinase domain. These bentgrass ESTs with hits from the *B. distachyon* proteome and homologous genes were localized mainly to *B. distachyon* chromosomes 1, 2 and 3. Chromosomes 1 and 3 of *B. distachyon* showed synteny with bentgrass LG7.1 where the major QTL for field resistance to dollar spot was found (Figure 5). Chakraborty et al. [22] detected additional dollar spot resistance QTLs with smaller effect on LGs 2.1, 3.2, 4.1–4.2, 6.2 and 7.2 that present synteny with *B. distachyon* chromosomes 1, 2 and 3. A recent study indicated a higher level of synteny and conservation of resistance gene positions between *B. distachyon* and barley, as compared with rice and barley [41]. Although rice has been extensively used as a model system for grasses, genome information of *B. distachyon* could be easily transferable for comparative studies, at least within Pooideae species due to evolutionary closeness. Future studies should explore the relationship between QTL for traits of interest in the creeping bentgrass linkage map and genes located in syntenic regions of *B. distachyon*.

In conclusion, the current genetic map of allotetraploid creeping bentgrass was, for the first time, used for comparative genome analysis with the Triticeae, ryegrass, oat, and rice based on a common set of EST-RFLP probes. This comparative map establishes a method for genome organization among bentgrass species as well as gives an understanding of the genome relationships of bentgrass with these Poaceae species. We found several large-scale chromosomal rearrangements between creeping bentgrass and the cereal crops. However, no evidence of large-scale chromosomal rearrangement was detected between two recently domesticated species, creeping bentgrass and ryegrass suggesting that these two species might be more closely related. In addition, the comparative map with *B. distachyon* would serve as a roadmap to develop PCR-based creeping bentgrass markers from a set of single-copy conserved orthologous genes in *Brachypodium* species.

Our future goal is to utilize genetic information from well-studied model species like rice, and now *B. distachyon,* through comparative mapping, to locate orthologous genes of traits of interest in bentgrass and eventually, to develop improved creeping bentgrass cultivars using classical and molecular breeding approaches. Currently, this creeping bentgrass map has served to detect a QTL for field resistance to dollar spot [22] and will be utilized for mapping QTLs and developing functional markers for important traits of interest in the golf industry such as drought, cold, heat, salt and disease tolerance, as well as leaf color, aggressiveness, and shoot density.

## Materials and Methods

### Linkage map construction

The previously published EST-RFLP marker data (derived from grass genome anchor probes, BCD, CDO, and RZ; from creeping bentgrass probes, Ast) of the 549×372 creeping bentgrass population (Table 1) [22] was used to reconstruct a genetic linkage map using JoinMap 3.0 software [42], which can handle outcrossing species using any marker types with differing modes of segregation. For example, analyzed RFLP markers were categorized into one of five segregation types: 1) a heterozygous locus in both parents with four alleles (ab x cd), 2) a heterozygous locus in both parents with one common allele (ef x eg), 3) a heterozygous locus in both parents with two common alleles (hk x hk), 4) a heterozygous locus in the maternal parent (lm x ll), and 5) a heterozygous locus in the paternal parent (nn x np). Logarithm of odds ratio (LOD) thresholds used for grouping markers ranged from 4 to 10. The numbering of each pair of homoeologous linkage groups (LGs) was followed to Chakraborty et al. [22] and map distances were calculated using the Kosambi mapping function [43].

### Comparative genome analysis

The bentgrass 549×372 EST-RFLP genetic map was compared with the consensus genetic map for Triticeae species developed among *T. aestivum*, *T. tauschii*, *H. vulgare*, and *H. spontaneum* (hereafter referred to as the Triticeae) available from the GrainGenes website (http://wheat.pw.usda.gov/ggpages/maps.shtml) [44]. Comparative map analysis with ryegrass was conducted based on a common set of RFLP markers using the previously published ryegrass maps [30], [32] and the recently updated ryegrass map mapped with 13 additional Ast probes [39]. Comparative map analysis with oat was performed through comparison with the oat map of Van Deynze et al. [12]. Comparative map analysis with rice was conducted using maps obtained from the Gramene website (http://www.gramene.org/cmap) and the rice map of Ahn and Tanksley [45]. For the purpose of the comparative genome analysis in this study, a syntenic block between the two species compared was defined when a segment containing two syntenic loci was not disrupted by two consecutive non-syntenic loci.

Mapped bentgrass EST-RFLP Ast probes were further analyzed for their putative function by blasting their sequences against protein sequences of known genes at an *e*-value ≤ 1×10^−5^ as a cut-off using BLASTX on the NCBI’s web server. Map locations of Ast probes were deduced from syntenic locations in rice chromosomes using sequence similarity with a *Japonica* rice cDNA probe collection [46] and the Triticeae synteny information [47; http://wheat.pw.usda.gov/pubs/2003/Sorrells]. Marker locations and the order detected with the common probes in each genetic map of the Triticeae, ryegrass, oat, and rice were represented according to the published information cited above.

Since the EST-RFLP markers mapped in the creeping bentgrass map do not have genetically mapped information in *B. distachyon*, the comparative analysis with this species was performed using BLASTN at the *B. distachyon* web sites (http://www.brachybase.org and http://www.phytozome.net) to determine the chromosomal location of the EST-RFLP markers from the creeping bentgrass linkage map by selecting the alignments with *e*-value ≤ 1×10^−10^. In addition, sequences of 8,470 *A. stolonifera* ESTs (AgEST) publicly available [33] were blasted against the *B. distachyon* genome to find orthologous loci between these two species. Before the BLAST analysis all the redundant AgEST sequences (2,511 sequences) were removed using the CD-HIT-EST program with a sequence identity cut off ≥ 90% [48]. The identification of orthologs was based on two more stringent parameters, Cumulative Identity Percentage (CIP) and Cumulative Alignment Length Percentage (CALP), and values of CIP ≥ 60% and CALP ≥ 70% were used as thresholds [8], [34].

## Supporting Information

Table S1 **Creeping bentgrass cDNA probes mapped on 14 linkage groups of the creeping bentgrass reference population (549 x 372)** [**22**] **.** The *e*-value of the cDNA sequence that matches to database (BLASTX) and the map position of the bentgrass ESTs relative to rice [46] and wheat [47] (http://wheat.pw.usda.gov/pubs/2003/Sorrells/) are indicated.
(DOCX)

Table S2 ***Brachypodium distachyon*** **chromosomal map position of sequences of EST-RFLP markers mapped on creeping bentgrass linkage map.**
(DOCX)

Table S3 **Creeping bentgrass ESTs orthologous to** ***Brachypodium distachyon*** **chromosomes.**
(DOCX)
