# Supplementary material for: Comparative Genome Analysis between Agrostis stolonifera and Members of the Pooideae Subfamily, including Brachypodium distachyon
Source: PLoS One. 2013 Nov 11;8(11):e79425. doi: 10.1371/journal.pone.0079425 (PMC3823605; doi:10.1371/journal.pone.0079425)
Supplement: Table S1 — Creeping bentgrass cDNA probes mapped on 14 linkage groups of the creeping bentgrass reference population (549 x 372) [22] . The e-value of the cDNA sequence that matches to database (BLASTX) and the map position of the bentgrass ESTs relative to rice [46] and wheat [47] (http://wheat.pw.usda.gov/pubs/2003/Sorrells/) are indicated. (DOCX) [file pone.0079425.s001.docx]

Table S1. Creeping bentgrass cDNA clones mapped on 14 linkage groups of the creeping bentgrass reference population (549 x 372) [18]. The *e*-value of the cDNA sequence that matches to database (BLASTX) and the map position of the bentgrass ESTs relative to rice [41] and wheat [43] (http://wheat.pw.usda.gov/pubs/2003/Sorrells/) are indicated.

| Bentgrass cDNA clone | Gene bank Accession ID | Putative function | *e*-value | Putative location on chromosomes | | Bentgrass LG |
| --- | --- | --- | --- | --- | --- | --- |
|  |  |  |  | Wheat | Rice |  |
| Ast5151 | DY543459 | putative U2 snRNP auxiliary factor [*Oryza sativa*] | 7e-08 | 4 | 5 | 1 |
| Ast54 | DY543383 | No significant similarity found. | - | 1 | 5 | 1 |
| Ast355 | DY543397 | phosphoglycerate kinase, putative [*Arabidopsis thaliana*] | 6e-09 | 1 | 5 | 1 |
| Ast3151 | DY543410 | unknown protein [*A. thaliana*] | 1e-40 | 1 | 5 | 1 |
| Ast5208 | DY543591 | glycine cleavage system H protein, mitochondrial, putative [*A. thaliana*] | 2e-63 | 1 | 10 | 1 |
| Ast39 | DY543370 | chitinase (EC 3.2.1.14) precursor [*Beta vulgaris*] | 2e-06 | 2 | 4 | 2 |
| Ast348 | DY543341 | No strong matches | - | - | - | 2 |
| Ast3102 | - | Mg-chelatase subunit XANTHA-F [*Hordeum vulgare*] | 2e-53 | 2 | 3 | 2 |
| Ast540 | DY543363 | homeobox-leucine zipper protein homolog [*Glycine max*] | 2e-14 | - | 4 | 2 |
| Ast5145 | DY543455 | ribosomal protein L12.1 precursor, chloroplast [*Secale cereale*] | 2e-53 | - | 7 | 2 |
| Ast580 | - | No hit | - | 2 | 4 | 2 |
| Ast567 | DY543447 | thylakoid lumen 15.0 kDa protein [*A. thaliana*] | 2e-39 | - | 7 | 2 |
| Ast5162 | DY543463 | chlorophyll A-B binding protein, chloroplast (LHCB6) [*A. thaliana*] | 1e-47 | 2 | 4 | 2 |
| Ast33 | DY543367 | No hit | - | - | 1 | 3 |
| Ast3277 | DY543641 | putative histidine kinase [*O. sativa*] | 1e-17 | 3 | 1 | 3 |
| Ast555 | DY543416 | oxygen-evolving enhancer protein 3-1, chloroplast precursor (16 kDa subunit photosystem II) [*O. sativa*] | 1e-45 | - | 1 | 3 |
| Ast564 | DY543445 | No hit | - | - | 1 | 3 |
| Ast571 | DY543450 | oat victorin binding protein [*Avena sativa*] | 5e-64 | 3 | 1 | 3 |
| Ast5166 | DY543467 | metallothionein-like protein type 3 [*H. vulgare*] | 6e-14 | 1 | 1 | 3 |
| Ast5170 | DY543421 | Aux/IAA protein [*Solanum tuberosum*] | 1e-34 | 1 | 1 | 3 |
| Ast388 | DY543471 | No hit | - | 4 | 3 | 4 |
| Ast349 | DY543394 | unknown protein [*O. sativa*] | 2e-11 | 4 | 3 | 4 |
| Ast572 | DY543451 | putative methylenetetrahydrofolate reductase [*O. sativa*] | e-117 | - | 3 | 4 |
| Ast546 | DY543365 | putative serine/threonine-specific protein kinase [*O. sativa*] | 3e-62 | 4 | 3 | 4 |
| Ast5103 | DY543488 | putative serine/threonine kinase [*O. sativa*] | 1e-43 | 4 | 3 | 4 |
| Ast5168 | - | No hit | - | - | 6 | 4 |
| Ast559 | DY543419 | nonspecific lipid transfer protein Cw-18 precursor - barley [*H. vulgare*] | 6e-27 | 2 | 12 | 4 |
| Ast53 | DY543382 | Serine hydroxymethyltransferase, mitoch. Precursor [*S. tuberosum*] | 0.0 | 4 | 3 | 4 |
| Ast357 | DY543398 | PS I 10.8 kDa reaction center subunit IV, chloroplast precursor (PSI-E) [*H. vulgare*] | 6e-21 | 4 | 7 | 5 |
| Ast323 | DY543373 | No hit | - | - | 9 | 5 |
| Ast3110 | - | No hit | - | 3 | 4 | 5 |
| Ast563 | DY543444 | fructose-bisphosphate aldolase (EC 4.1.2.13) [*O. sativa*] | 4e-47 | 5 | 11 | 5 |
| Ast3163 | DY543439 | expressed protein [*A. thaliana*] | 3e-59 | 1 | 2 | 6 |
| Ast3115 | DY543486 | putative brown planthopper susceptibility protein Hd002A [*O. sativa*] | 2e-34 | - | 2 | 6 |
| Ast3199 | DY543537 | chloroplast translational elongation factor Tu [*O. sativa*] | e-115 | 6 | 2 | 6 |
| Ast528 | DY543387 | putative WRKY9-10 protein [*H. vulgare*] | 4e-05 | 6 | 2 | 6 |
| Ast529 | DY543388 | phosphoribulokinase; ribulose-5-phosphate kinase [*T. aestivum*] | 2e-57 | 7 | 2 | 6 |
| Ast32 | DY543366 | putative auxin-regulated protein [*O. sativa*] | 2e-54 | 1 | 8 | 7 |
| Ast369 | DY543427 | ALDH2b mRNA for mitochondrial aldehyde dehydrogenase [*S. cereale*] | 0.0 | - | - | 7 |
| Ast3213 | DY543567 | chlorophyll a/b binding protein precursor [*H. vulgare*] | 2e-97 | - | 6 | 7 |
| Ast56 | DY543344 | putative UOS1 [*O. sativa*] (Pred. nucleoside-diphosphate-sugar epimerases: weaker) | 5e-96 | 7 | 6 | 7 |
| Ast533 | DY543389 | knotted 6 [*H. vulgare*] / Putative homeobox gene [*O. sativa*] | 3e-60 | 7 | 6 | 7 |
| Ast554 | DY543415 | vacuolar proton-inorganic pyrophosphatase [*H. brevisubulatum*] | 3e-58 | 7 | 6 | 7 |
| Ast5381 | DY543790 | Ras-related GTP-binding family protein [*A. thaliana*] | 4e-50 | - | 6 | 7 |
| Ast5213 | DY543594 | putative cytochrome B5 [*O. sativa*]; susceptibility homeodomain transcription factor [*O. sativa*] | 1e-43 | 3 | 6 | 7 |
| Ast568 | DY543448 | putative MATE efflux family protein [*O. sativa*] | 3e-82 | 3 | 3 | 7 |
| Ast574 | DY543452 | No hit | - | - | 6 | 7 |
| Ast5150 | DY543458 | nucleosidase-related [*A. thaliana*] | 5e-22 | - | 1 | 7 |
| Ast5115 | DY543497 | putative protein kinase [*O. sativa*] | 1e-23 | - | 6 | 7 |
