# Supplementary material for: Comparative Genome Analysis between Agrostis stolonifera and Members of the Pooideae Subfamily, including Brachypodium distachyon
Source: PLoS One. 2013 Nov 11;8(11):e79425. doi: 10.1371/journal.pone.0079425 (PMC3823605; doi:10.1371/journal.pone.0079425)
Supplement: Table S2 — Brachypodium distachyon chromosomal map position of sequences of EST-RFLP markers mapped on creeping bentgrass linkage map. (DOCX) [file pone.0079425.s002.docx]

Table S2. *B. distachyon* chromosome location of sequences of EST-RFLP markers mapped on genetic map of creeping bentgrass.

| Bentgrass LG ^a^ | Marker (Gene Bank Accession ID) ^b^ | Bd chromosome ^c^ | Bd chromosome location ^d^ | |
| --- | --- | --- | --- | --- |
|  |  |  | Start | End |
| 1.2 | CDO202 | 2 | 13312741 | 13313054 |
| 1.1-1.2 | BCD1261 | 2 | 13918752 | 13919099 |
| 1.1-1.2 | CDO1160 | 2 | 13959438 | 13959911 |
| 1.1 | Ast5244 (DY543632) | 2 | 17734568 | 17737309 |
| 1.2 | BCD738 | 2 | 18485474 | 18485955 |
| 1.2 | Ast355 (DY543397) | 2 | 18485698 | 18485954 |
| 1.2 | CDO89 | 2 | 19921391 | 19922005 |
| 1.2 | Ast3151 (DY543410) | 2 | 22117132 | 22119285 |
| 1.1-1.2 | CDO771 | 2 | 22596648 | 22597674 |
| 1.1 | BCD454 | 2 | 23921191 | 23921611 |
| 1.1-1.2 | BCD1072 | 2 | 30224103 | 30224321 |
| 1.1 | BCD200 | 2 | 30230242 | 30230609 |
| 1.2 | Ast5185 (DY543548) | 2 | 36219723 | 36221116 |
| 1.1 | CDO580 | 2 | 38466405 | 38467009 |
| 1.2 | RZ444 | 2 | 53296932 | 53297140 |
| 1.2 | BCD921 | 3 | 20772298 | 20772621 |
| 1.1 | CDO98 | 3 | 30319151 | 30321086 |
| 1.2 | BCD386 | 3 | 32086894 | 32087540 |
| 1.2 | Ast5208 (DY543591) | 3 | 32651725 | 32653364 |
| 1.2 | CDO94 | 3 | 32721646 | 32722742 |
| 2.1 | BCD1184 | 1 | 13478584 | 13478963 |
| 2.1-2.2 | Ast552 (DY543414) | 1 | 14595910 | 14600036 |
| 2.1-2.2 | Ast5145 (DY543455) | 1 | 14908782 | 14909109 |
| 2.1-2.2 | Ast3102 ^e^ | 1 | 15401288 | 15401827 |
| 2.1-2.2 | CDO405 | 1 | 15743376 | 15743751 |
| 2.1-2.2 | BCD1709 | 1 | 19259600 | 19259999 |
| 2.1-2.2 | CDO59 | 1 | 20953161 | 20953582 |
| 2.1 | BCD1 | 1 | 23483880 | 23484122 |
| 2.1 | Ast354 (DY543396) | 1 | 24105397 | 24107492 |
| 2.1 | BCD438 | 1 | 53957117 | 53957391 |
| 2.1 | BCD1184 | 1 | 64356335 | 64357924 |
| 2.2 | CDO365 | 4 | 17638266 | 17638709 |
| 2.2 | Ast567 (DY543447) | 5 | 4337487 | 4339261 |
| 2.1-2.2 | Ast5162 (DY543463) | 5 | 15617190 | 15617775 |
| 2.1 | CDO1328 | 5 | 15844066 | 15847157 |
| 2.1-2.2 | CDO684 | 5 | 21100968 | 21101188 |
| 3.2 | **Ast563** (DY543444) | 2 | 841745 | 842266 |
| 3.1-3.2 | Ast5166 (DY543467) | 2 | 4664467 | 4665522 |
| 3.2 | Ast5170 (DY543421) | 2 | 6119943 | 6123643 |
| 3.1 | CDO328 | 2 | 6867698 | 6868202 |
| 3.2 | BCD127 | 2 | 8725766 | 8726125 |
| 3.1-3.2 | RZ995 | 2 | 9220956 | 9221418 |
| 3.1 | BCD1495 | 2 | 9496671 | 9497017 |
| 3.1 | BCD1495 | 2 | 10491102 | 10491445 |
| 3.1 | BCD1495 | 2 | 10495830 | 10496174 |
| 3.1-3.2 | BCD134 | 2 | 41622340 | 41623472 |
| 3.1-3.2 | BCD828 | 2 | 47227872 | 47228154 |
| 3.2 | CDO1174 | 2 | 47437527 | 47437914 |
| 3.1 | Ast571 (DY543450) | 2 | 48360330 | 48361363 |
| 3.1-3.2 | Ast3277 (DY543641) | 2 | 56909915 | 56911116 |
| 3.1-3.2 | Ast3277 (DY543641) | 2 | 58867188 | 58867992 |
| 3.1-3.2 | Ast3277 (DY543641) | 2 | 58868700 | 58869561 |
| 3.1 | BCD98 | 3 | 44128540 | 44129179 |
| 3.2 | CDO99 | 3 | 44130090 | 44130609 |
| 3.2 | CDO585 | 4 | 7093558 | 7093796 |
| 3.2 | **Ast563** (DY543444) | 4 | 29522820 | 29523351 |
| 3.1-3.2 | CDO244 | 5 | 24490389 | 24490639 |
| 4.1-4.2 | CDO484 | 1 | 1366964 | 1367495 |
| 4.1 | Ast572 (DY543451) | 1 | 2811156 | 2813423 |
| 4.1 | Ast349 (DY543394) | 1 | 3103523 | 3104213 |
| 4.2 | BCD808 | 1 | 4998784 | 4999139 |
| 4.1-4.2 | CDO504 | 1 | 5962259 | 5962873 |
| 4.2 | BCD450 | 1 | 5988342 | 5988796 |
| 4.2 | BCD808 | 1 | 7318997 | 7319438 |
| 4.1-4.2 | CDO542 | 1 | 8350647 | 8351378 |
| 4.1 | CDO122 | 1 | 8567153 | 8567563 |
| 4.1-4.2 | CDO795 | 1 | 11769377 | 11770588 |
| 4.1-4.2 | BCD385 | 1 | 12074984 | 12075099 |
| 4.2 | BCD808 | 1 | 19135955 | 19136422 |
| 4.2 | CDO938 | 1 | 62486394 | 62487017 |
| 4.2 | CDO38 | 1 | 62730415 | 62730828 |
| 4.2 | Ast5103 (DY543488) | 1 | 63213682 | 63215164 |
| 4.2 | CDO1395 | 1 | 68017621 | 68018050 |
| 4.1-4.2 | Ast3325 (DY543730) | 1 | 68060410 | 68061124 |
| 4.1-4.2 | CDO1401 | 1 | 71738939 | 71739260 |
| 4.1-4.2 | CDO20 | 1 | 72945681 | 72946010 |
| 4.1-4.2 | CDO20 | 1 | 72951260 | 72951645 |
| 4.1 | CDO241 | 4 | 11555301 | 11555602 |
| 4.1 | CDO520 | 4 | 14398184 | 14398326 |
| 4.2 | Ast559 (DY543419) | 4 | 31093780 | 31094131 |
| 5.1 | Ast357 (DY543398) | 1 | 24421682 | 24422146 |
| 5.2 | **CDO1049** | 3 | 19476497 | 19477936 |
| 5.1-5.2 | Ast5343 (DY543747) | 3 | 21176323 | 21178461 |
| 5.1-5.2 | Ast551 (DY543413) | 3 | 21620907 | 21621429 |
| 5.1 | CDO749 | 4 | 1558290 | 1559232 |
| 5.1-5.2 | CDO677 | 4 | 2023698 | 2024300 |
| 5.1 | CDO344 | 4 | 3112832 | 3113225 |
| 5.1 | Ast394 ^e^ | 4 | 6307762 | 6308465 |
| 5.2 | **CDO1049** | 4 | 7497676 | 7500061 |
| 5.1 | CDO344 | 4 | 8453360 | 8453651 |
| 5.1 | CDO344 | 4 | 8457255 | 8457560 |
| 5.1 | CDO344 | 4 | 8460278 | 8460619 |
| 5.1 | CDO344 | 4 | 8464909 | 8465214 |
| 5.1 | CDO344 | 4 | 8471117 | 8471457 |
| 5.1 | CDO344 | 4 | 8474194 | 8474511 |
| 5.1 | CDO344 | 4 | 8480368 | 8480693 |
| 5.1 | CDO344 | 4 | 8483793 | 8484112 |
| 5.1 | CDO344 | 4 | 8486603 | 8486922 |
| 5.1 | CDO344 | 4 | 8493131 | 8493419 |
| 5.1 | CDO344 | 4 | 8497047 | 8497331 |
| 5.1 | CDO344 | 4 | 8502867 | 8503174 |
| 5.1 | CDO344 | 4 | 8530596 | 8530922 |
| 5.1 | CDO344 | 4 | 8539446 | 8539781 |
| 5.1 | CDO344 | 4 | 8544334 | 8544651 |
| 5.1 | CDO344 | 4 | 8547916 | 8548212 |
| 5.1-5.2 | Ast563 (DY543444) | 4 | 29522820 | 29523351 |
| 5.2 | BCD1087 | 4 | 33816927 | 33817270 |
| 5.1-5.2 | CDO989 | 4 | 36917718 | 36917857 |
| 5.1-5.2 | CDO412 | 4 | 37681112 | 37681669 |
| 5.1 | BCD1088 | 4 | 38888244 | 38888602 |
| 5.2 | CDO127 | 4 | 46698455 | 46698943 |
| 6.1-6.2 | **CDO1158** | 1 | 27070854 | 27071168 |
| 6.1 | **CDO686** | 1 | 42666761 | 42667080 |
| 6.1-6.2 | **CDO1158** | 3 | 3998204 | 3998548 |
| 6.1-6.2 | CDO534 | 3 | 5115466 | 5116010 |
| 6.1 | Ast3163 (DY543439) | 3 | 48111250 | 48114561 |
| 6.1-6.2 | CDO497 | 3 | 49118762 | 49119005 |
| 6.2 | Ast3199 (DY543537) | 3 | 49305661 | 49306768 |
| 6.1-6.2 | CDO1380 | 3 | 49359330 | 49359740 |
| 6.1 | Ast370 (DY543428) | 3 | 50900443 | 50900807 |
| 6.1-6.2 | BCD1860 | 3 | 53533432 | 53533752 |
| 6.2 | RZ273 | 3 | 54188671 | 54188821 |
| 6.2 | CDO507 | 3 | 54190265 | 54190547 |
| 6.2 | Ast3115 (DY543486) | 3 | 55685645 | 55686559 |
| 6.1-6.2 | Ast3155 ^e^ | 3 | 56863663 | 56864855 |
| 6.1 | CDO516 | 3 | 57450800 | 57451023 |
| 6.1-6.2 | **CDO686** | 3 | 58868011 | 58868332 |
| 6.1 | Ast5165 (DY543466) | 4 | 45075939 | 45076791 |
| 7.1-7.2 | Ast568 (DY543448) | 1 | 12573959 | 12576486 |
| 7.1 | **RZ508** | 1 | 25302337 | 25302576 |
| 7.1 | Ast554 (DY543415) | 1 | 25880365 | 25881182 |
| 7.1-7.2 | RZ682 | 1 | 26511824 | 26511978 |
| 7.1 | CDO673 | 1 | 33021887 | 33022102 |
| 7.1-7.2 | Ast5381 (DY543790) | 1 | 34370529 | 34371141 |
| 7.1 | **Ast5213** (DY543594) | 1 | 35552937 | 35553492 |
| 7.1 | Ast3213 (DY543567) | 1 | 39989003 | 39990068 |
| 7.1-7.2 | CDO17 | 1 | 40844693 | 40844982 |
| 7.1-7.2 | Ast369 (DY543427) | 1 | 41580006 | 41581370 |
| 7.2 | Ast5115 (DY543497) | 1 | 46751576 | 46751864 |
| 7.1 | CDO545 | 1 | 49180863 | 49181176 |
| 7.1 | RZ516 | 1 | 49336575 | 49336816 |
| 7.2 | BCD93 | 1 | 50094042 | 50094446 |
| 7.1 | **RZ508** | 1 | 72954229 | 72954468 |
| 7.2 | Ast5150 (DY543458) | 2 | 6103348 | 6103548 |
| 7.1 | **Ast5213** (DY543594) | 2 | 24920787 | 24921343 |
| 7.1 | **RZ508** | 3 | 928721 | 928957 |
| 7.1 | BCD349 | 3 | 11439298 | 11439995 |
| 7.1 | BCD276 | 3 | 38400248 | 38401418 |
| 7.1-7.2 | Ast5121 (DY543501) | 3 | 39274997 | 39275975 |
| 7.1 | CDO464 | 3 | 41043884 | 41046423 |
| 7.1 | CDO595 | 3 | 42643624 | 42643830 |
| 7.2 | BCD98 | 3 | 44128540 | 44129179 |
| 7.2 | CDO99 | 3 | 44130090 | 44130609 |

^a^ Creeping bentgrass linkage group (LG) where the marker was mapped.

^b^ The markers in bold correspond to those that matched in more than one *B.* *distachyon* chromosome.

^c^ *B.* *distachyon* chromosome where the marker was found after the BLASTN search.

^d^ Location of the alignment between the marker and the *B.* *distachyon* chromosome (start and end correspond to base pair number).

^e^ Sequence not available at NCBI.
