# Supplementary material for: Comparative Genome Analysis between Agrostis stolonifera and Members of the Pooideae Subfamily, including Brachypodium distachyon
Source: PLoS One. 2013 Nov 11;8(11):e79425. doi: 10.1371/journal.pone.0079425 (PMC3823605; doi:10.1371/journal.pone.0079425)
Supplement: Table S3 — Creeping bentgrass ESTs orthologous to Brachypodium distachyon chromosomes. (DOCX) [file pone.0079425.s003.docx]

Table S3. Creeping bentgrass EST orthologs to *B. distachyon* chromosomes.

| *Agrostis*  EST ID | Bd  chromosome ^b^ | Bd chromosome location ^a^ | | Duplications | |
| --- | --- | --- | --- | --- | --- |
|  |  | Start | End | Within (#) ^c^ | Among (Bdch) ^d^ |
| DV860292 | 1 | 58122365 | 58122254 |  |  |
| DV860299 | 1 | 44145666 | 44145859 |  |  |
| DV860408 | 1 | 48045416 | 48045508 |  |  |
| DV860460 | 1 | 5767669 | 5767618 |  |  |
| DV860498 | 1 | 20235456 | 20235377 |  |  |
| DV860500 | 1 | 64837233 | 64838211 |  | Bd3 |
| DV860573 | 1 | 4696595 | 4696055 | 3 | Bd4 |
| DV860604 | 1 | 69418662 | 69418945 |  |  |
| DV860646 | 1 | 18654987 | 18654820 |  |  |
| DV860829 | 1 | 45677406 | 45677354 |  |  |
| DV860890 | 1 | 60661123 | 60661243 |  |  |
| DV860997 | 1 | 50443381 | 50443446 |  |  |
| DV861094 | 1 | 61951160 | 61951233 |  |  |
| DV861115 | 1 | 58331771 | 58331708 |  |  |
| DV861154 | 1 | 5190381 | 5190651 |  |  |
| DV861286 | 1 | 9087868 | 9087689 |  |  |
| DV861287 | 1 | 7148045 | 7148191 |  |  |
| DV861317 | 1 | 8149957 | 8150325 |  |  |
| DV861320 | 1 | 50081458 | 50081603 |  |  |
| DV861326 | 1 | 45677260 | 45677197 |  |  |
| DV861401 | 1 | 16199480 | 16198922 | 2 |  |
| DV861438 | 1 | 5190249 | 5190675 |  |  |
| DV861460 | 1 | 13812451 | 13812769 |  |  |
| DV861483 | 1 | 74281932 | 74281970 |  |  |
| DV861541 | 1 | 4094647 | 4094675 |  |  |
| DV861568 | 1 | 13812524 | 13812769 |  |  |
| DV861577 | 1 | 70726749 | 70726604 |  |  |
| DV861613 | 1 | 72499792 | 72499653 |  |  |
| DV861633 | 1 | 63754352 | 63754049 |  |  |
| DV861655 | 1 | 7976981 | 7976902 |  |  |
| DV861676 | 1 | 62732883 | 62732736 |  |  |
| DV861700 | 1 | 48423826 | 48424626 |  |  |
| DV861736 | 1 | 21906435 | 21906124 |  |  |
| DV861802 | 1 | 33222172 | 33221876 |  |  |
| DV861828 | 1 | 9773669 | 9773516 |  |  |
| DV861866 | 1 | 63094796 | 63094718 |  |  |
| DV861915 | 1 | 63607740 | 63607830 |  |  |
| DV862048 | 1 | 9019073 | 9019421 |  | Bd2, 3, 4 |
| DV862055 | 1 | 66614079 | 66614109 |  |  |
| DV862135 | 1 | 4109009 | 4109080 |  |  |
| DV862142 | 1 | 61924029 | 61923976 | 2 |  |
| DV862161 | 1 | 20665137 | 20664943 |  |  |
| DV862168 | 1 | 1827223 | 1827426 |  |  |
| DV862191 | 1 | 21169471 | 21169737 |  |  |
| DV862253 | 1 | 69507164 | 69506853 |  |  |
| DV862293 | 1 | 14550469 | 14550194 |  |  |
| DV862417 | 1 | 40736060 | 40735806 | 3 | Bd2, 3, 4, 5 |
| DV862436 | 1 | 62984506 | 62984239 |  |  |
| DV862455 | 1 | 8567980 | 8567530 |  |  |
| DV862491 | 1 | 15300725 | 15300920 |  |  |
| DV862560 | 1 | 65870991 | 65870793 |  |  |
| DV862581 | 1 | 27643540 | 27643518 |  |  |
| DV862694 | 1 | 17151824 | 17151560 |  |  |
| DV862801 | 1 | 50540315 | 50540420 |  |  |
| DV862836 | 1 | 7703418 | 7703152 |  |  |
| DV862864 | 1 | 6390655 | 6390506 |  |  |
| DV862946 | 1 | 14212470 | 14212518 |  |  |
| DV862967 | 1 | 28326959 | 28327251 | 3 | Bd2 |
| DV863053 | 1 | 12482891 | 12483277 |  |  |
| DV863161 | 1 | 4167967 | 4167607 |  | Bd5 |
| DV863251 | 1 | 3337557 | 3337358 |  |  |
| DV863289 | 1 | 41623175 | 41623058 |  |  |
| DV863339 | 1 | 54705284 | 54705750 |  |  |
| DV863383 | 1 | 50093974 | 50094303 |  |  |
| DV863417 | 1 | 68743290 | 68743522 |  |  |
| DV863428 | 1 | 62356412 | 62356589 |  |  |
| DV863533 | 1 | 8312570 | 8312298 |  |  |
| DV863568 | 1 | 57063707 | 57063877 |  |  |
| DV863585 | 1 | 27644182 | 27644136 |  |  |
| DV863610 | 1 | 68584591 | 68584504 |  |  |
| DV863711 | 1 | 41875484 | 41875567 |  |  |
| DV863776 | 1 | 68237345 | 68237305 |  |  |
| DV863894 | 1 | 66404574 | 66404632 |  |  |
| DV863905 | 1 | 7319394 | 7318933 |  |  |
| DV863972 | 1 | 63265235 | 63265356 |  |  |
| DV863977 | 1 | 70427108 | 70426975 |  |  |
| DV864001 | 1 | 31324229 | 31324410 |  |  |
| DV864040 | 1 | 1827016 | 1827060 |  |  |
| DV864053 | 1 | 20665860 | 20665690 |  |  |
| DV864078 | 1 | 61924016 | 61923976 |  |  |
| DV864090 | 1 | 65468808 | 65468574 |  |  |
| DV864141 | 1 | 66405253 | 66404970 |  |  |
| DV864224 | 1 | 67130227 | 67130126 |  |  |
| DV864253 | 1 | 70031121 | 70030905 |  |  |
| DV864353 | 1 | 15392991 | 15393057 |  |  |
| DV864466 | 1 | 62192267 | 62192146 |  |  |
| DV864484 | 1 | 65941385 | 65941133 |  |  |
| DV864629 | 1 | 58231019 | 58230905 |  |  |
| DV864659 | 1 | 61653588 | 61653529 |  |  |
| DV864674 | 1 | 24421769 | 24421956 |  |  |
| DV864721 | 1 | 50442967 | 50443035 |  |  |
| DV864795 | 1 | 67267510 | 67267295 |  |  |
| DV864836 | 1 | 68520537 | 68520642 |  |  |
| DV864856 | 1 | 32343727 | 32343608 |  |  |
| DV864873 | 1 | 64721655 | 64721282 |  |  |
| DV864877 | 1 | 53393692 | 53393817 |  |  |
| DV864895 | 1 | 33459406 | 33459594 |  |  |
| DV864903 | 1 | 34368864 | 34368949 |  |  |
| DV864919 | 1 | 20234724 | 20234779 |  |  |
| DV864929 | 1 | 5005786 | 5005835 |  |  |
| DV864935 | 1 | 64592884 | 64593196 |  |  |
| DV865001 | 1 | 32280141 | 32280519 |  |  |
| DV865003 | 1 | 68683540 | 68683948 |  |  |
| DV865043 | 1 | 28915490 | 28915411 |  |  |
| DV865075 | 1 | 1867227 | 1867175 |  |  |
| DV865098 | 1 | 6677073 | 6677035 |  |  |
| DV865127 | 1 | 46551540 | 46551366 |  |  |
| DV865133 | 1 | 7647707 | 7647819 |  |  |
| DV865195 | 1 | 8341195 | 8341289 |  |  |
| DV865392 | 1 | 10068223 | 10068158 |  | Bd4 |
| DV865471 | 1 | 62627778 | 62627626 |  |  |
| DV865532 | 1 | 62984068 | 62983804 |  |  |
| DV865533 | 1 | 15415853 | 15415740 |  |  |
| DV865669 | 1 | 54824816 | 54824718 |  |  |
| DV865674 | 1 | 54728480 | 54727963 | 2 | Bd3 |
| DV865696 | 1 | 64744478 | 64744433 |  |  |
| DV865701 | 1 | 23554411 | 23553534 |  | Bd3, 5 |
| DV865708 | 1 | 54728507 | 54727963 | 2 | Bd3 |
| DV865724 | 1 | 20997621 | 20996942 |  |  |
| DV865739 | 1 | 6796569 | 6797013 |  |  |
| DV865785 | 1 | 66593521 | 66593487 |  |  |
| DV865804 | 1 | 54728502 | 54727963 | 2 | Bd3 |
| DV865826 | 1 | 54728480 | 54727963 | 2 |  |
| DV865841 | 1 | 72877071 | 72876983 |  |  |
| DV865868 | 1 | 69647995 | 69647889 |  |  |
| DV865884 | 1 | 8115004 | 8115227 |  | Bd2, 3, 4 |
| DV865886 | 1 | 43151697 | 43151965 |  |  |
| DV865950 | 1 | 41875866 | 41875939 |  |  |
| DV865964 | 1 | 3828360 | 3828166 |  |  |
| DV865986 | 1 | 19136181 | 19135848 |  |  |
| DV866003 | 1 | 72138556 | 72138667 |  |  |
| DV866026 | 1 | 17183424 | 17183235 |  |  |
| DV866076 | 1 | 25391399 | 25391539 |  |  |
| DV866214 | 1 | 29785607 | 29785382 |  |  |
| DV866247 | 1 | 20109516 | 20109787 |  |  |
| DV866274 | 1 | 44341630 | 44341496 |  |  |
| DV866364 | 1 | 3217278 | 3217478 |  |  |
| DV866367 | 1 | 5802618 | 5802512 |  |  |
| DV866392 | 1 | 74078223 | 74078355 |  |  |
| DV866394 | 1 | 6819372 | 6819658 |  |  |
| DV866507 | 1 | 14019536 | 14019453 |  |  |
| DV866687 | 1 | 30385706 | 30385357 |  |  |
| DV866702 | 1 | 35760109 | 35759575 | 3 | Bd3, 5 |
| DV866776 | 1 | 31324341 | 31324410 |  |  |
| DV866824 | 1 | 65468866 | 65468574 |  |  |
| DV866921 | 1 | 8340282 | 8340361 |  | Bd4 |
| DV866922 | 1 | 44166696 | 44166921 |  |  |
| DV866929 | 1 | 2149343 | 2149313 |  |  |
| DV867049 | 1 | 6966390 | 6966466 |  |  |
| DV867162 | 1 | 10462049 | 10462189 |  |  |
| DV867180 | 1 | 68584555 | 68584504 |  |  |
| DV867204 | 1 | 69506655 | 69506731 |  |  |
| DV867236 | 1 | 16199534 | 16198927 | 2 |  |
| DV867322 | 1 | 55153808 | 55153276 |  |  |
| DV867327 | 1 | 62269705 | 62269766 |  |  |
| DV867350 | 1 | 66515083 | 66515016 |  |  |
| DV867402 | 1 | 18722397 | 18722044 |  |  |
| DV867410 | 1 | 44107086 | 44106533 |  |  |
| DV867431 | 1 | 3846565 | 3846703 |  |  |
| DV867599 | 1 | 22258140 | 22257826 |  |  |
| DV867624 | 1 | 3033162 | 3032946 |  |  |
| DV867642 | 1 | 50442963 | 50443044 |  |  |
| DV867821 | 1 | 22255772 | 22255637 |  |  |
| DV867864 | 1 | 65333302 | 65332849 | 2 | Bd2, 4 |
| DV867906 | 1 | 64232845 | 64232686 |  |  |
| DV867939 | 1 | 69319357 | 69319205 |  | Bd2 |
| DV867983 | 1 | 8332686 | 8332424 |  |  |
| DV868011 | 1 | 65871029 | 65870796 |  |  |
| DV868178 | 1 | 35676838 | 35677115 |  |  |
| DV868185 | 1 | 64232480 | 64232170 |  |  |
| DV868389 | 1 | 61315864 | 61315929 |  |  |
| DV868473 | 1 | 30732130 | 30732203 |  |  |
| DV868474 | 1 | 5005531 | 5005619 |  |  |
| DV868568 | 1 | 21392853 | 21393410 |  |  |
| DV868569 | 1 | 47050436 | 47050913 |  | Bd2 |
| DV868571 | 1 | 8567287 | 8567477 |  |  |
| DV868589 | 1 | 9106557 | 9106458 |  |  |
| DV868657 | 1 | 16184528 | 16184225 |  |  |
| DV868673 | 1 | 46551481 | 46551366 |  |  |
| DV868696 | 1 | 35767393 | 35767306 |  | Bd3 |
| DV860440 | 2 | 58828213 | 58828652 |  |  |
| DV860462 | 2 | 8130081 | 8130213 |  |  |
| DV860544 | 2 | 1942380 | 1942420 |  |  |
| DV860576 | 2 | 34715619 | 34715470 |  |  |
| DV860677 | 2 | 44943932 | 44943639 |  |  |
| DV860798 | 2 | 54950742 | 54950566 |  |  |
| DV860863 | 2 | 54885244 | 54884948 |  |  |
| DV861137 | 2 | 22735692 | 22736079 |  |  |
| DV861142 | 2 | 49899123 | 49899365 |  |  |
| DV861250 | 2 | 20686994 | 20687069 |  |  |
| DV861296 | 2 | 45586147 | 45586321 |  |  |
| DV861316 | 2 | 9053108 | 9053172 |  |  |
| DV861558 | 2 | 21810268 | 21810563 |  |  |
| DV861598 | 2 | 48682792 | 48682886 |  |  |
| DV861788 | 2 | 14654541 | 14654289 |  |  |
| DV861816 | 2 | 52560350 | 52560458 |  |  |
| DV861860 | 2 | 15745740 | 15745959 |  |  |
| DV861913 | 2 | 44385570 | 44385704 |  |  |
| DV862008 | 2 | 50520435 | 50520160 |  |  |
| DV862034 | 2 | 57071793 | 57071582 |  |  |
| DV862047 | 2 | 45333824 | 45333608 |  |  |
| DV862048 | 2 | 14366890 | 14366544 |  | Bd1, 3, 4 |
| DV862081 | 2 | 42711075 | 42710606 |  |  |
| DV862120 | 2 | 45267064 | 45267100 |  |  |
| DV862122 | 2 | 53025570 | 53025669 |  |  |
| DV862323 | 2 | 47926307 | 47926464 |  |  |
| DV862342 | 2 | 39007020 | 39007129 |  |  |
| DV862417 | 2 | 27605443 | 27605162 |  | Bd1, 3, 4, 5 |
| DV862428 | 2 | 48361001 | 48361277 |  |  |
| DV862475 | 2 | 25704451 | 25704594 |  |  |
| DV862658 | 2 | 34973349 | 34973455 |  |  |
| DV862727 | 2 | 42772559 | 42772621 |  |  |
| DV862859 | 2 | 27937279 | 27937380 |  |  |
| DV862967 | 2 | 34823412 | 34823373 |  | Bd1 |
| DV862973 | 2 | 42699139 | 42698868 |  | Bd4, 5 |
| DV862975 | 2 | 31967633 | 31967706 |  |  |
| DV863075 | 2 | 10775828 | 10776170 |  |  |
| DV863175 | 2 | 79191 | 79132 |  |  |
| DV863230 | 2 | 54913122 | 54913017 |  |  |
| DV863397 | 2 | 4131594 | 4131919 |  |  |
| DV863415 | 2 | 48702669 | 48702487 |  |  |
| DV863483 | 2 | 9426644 | 9426437 |  |  |
| DV863513 | 2 | 51187722 | 51187665 |  |  |
| DV863731 | 2 | 13885525 | 13885704 |  |  |
| DV863768 | 2 | 23277511 | 23277543 |  |  |
| DV863834 | 2 | 52751022 | 52751229 |  |  |
| DV863912 | 2 | 41541235 | 41541312 |  |  |
| DV864036 | 2 | 14654768 | 14654655 |  |  |
| DV864079 | 2 | 4874407 | 4873819 |  |  |
| DV864101 | 2 | 56324416 | 56324554 |  |  |
| DV864179 | 2 | 21525833 | 21526263 |  |  |
| DV864372 | 2 | 25387498 | 25387430 |  |  |
| DV864419 | 2 | 26128710 | 26128637 |  |  |
| DV864452 | 2 | 34559406 | 34559520 |  |  |
| DV864519 | 2 | 79103 | 78833 |  |  |
| DV864523 | 2 | 36805299 | 36805225 |  |  |
| DV864592 | 2 | 5535433 | 5535613 | 9 | Bd3, 4, 5 |
| DV864635 | 2 | 54698355 | 54698422 |  |  |
| DV864651 | 2 | 53521548 | 53521800 |  |  |
| DV864662 | 2 | 8647615 | 8647514 |  |  |
| DV864717 | 2 | 56324456 | 56324554 |  |  |
| DV864728 | 2 | 52517704 | 52517931 |  |  |
| DV864805 | 2 | 22829085 | 22828968 |  |  |
| DV864807 | 2 | 50017778 | 50017805 |  |  |
| DV864815 | 2 | 52389674 | 52389726 |  |  |
| DV864816 | 2 | 35174238 | 35174298 |  |  |
| DV864916 | 2 | 17396311 | 17396249 |  |  |
| DV864951 | 2 | 46311067 | 46310901 |  |  |
| DV864983 | 2 | 37775200 | 37775080 |  |  |
| DV865067 | 2 | 15854897 | 15854979 |  | Bd5 |
| DV865205 | 2 | 52015374 | 52015014 | 2 |  |
| DV865327 | 2 | 23489395 | 23489561 |  |  |
| DV865346 | 2 | 7157796 | 7157879 |  |  |
| DV865350 | 2 | 20681751 | 20681892 | 2 |  |
| DV865377 | 2 | 18485150 | 18485260 |  |  |
| DV865535 | 2 | 17737977 | 17737908 |  |  |
| DV865704 | 2 | 18328549 | 18328731 |  |  |
| DV865745 | 2 | 17737309 | 17737175 |  |  |
| DV865828 | 2 | 14730938 | 14730897 |  |  |
| DV865858 | 2 | 45333820 | 45333608 |  |  |
| DV865884 | 2 | 46083720 | 46083505 | 4 | Bd1, 3, 4 |
| DV865915 | 2 | 55881898 | 55881959 |  |  |
| DV865926 | 2 | 48854619 | 48854284 |  |  |
| DV866059 | 2 | 47349902 | 47349872 |  |  |
| DV866122 | 2 | 56980647 | 56980899 |  |  |
| DV866138 | 2 | 13931114 | 13931197 |  |  |
| DV866190 | 2 | 34514569 | 34514488 |  |  |
| DV866201 | 2 | 58846528 | 58846752 |  |  |
| DV866217 | 2 | 34715333 | 34715190 |  |  |
| DV866231 | 2 | 56169106 | 56169003 |  |  |
| DV866278 | 2 | 819649 | 820190 | 2 |  |
| DV866368 | 2 | 48359974 | 48360036 |  |  |
| DV866408 | 2 | 35945581 | 35945804 |  |  |
| DV866416 | 2 | 47445494 | 47445763 |  |  |
| DV866460 | 2 | 6723355 | 6723285 |  |  |
| DV866463 | 2 | 25704620 | 25704893 |  |  |
| DV866611 | 2 | 51077282 | 51077167 |  |  |
| DV866712 | 2 | 48360330 | 48360423 |  |  |
| DV866724 | 2 | 53137067 | 53137472 |  |  |
| DV866729 | 2 | 40629580 | 40630011 |  |  |
| DV866808 | 2 | 20797998 | 20797882 |  |  |
| DV866845 | 2 | 18329267 | 18329053 |  |  |
| DV866876 | 2 | 35296115 | 35296479 |  |  |
| DV867055 | 2 | 10495784 | 10495399 |  |  |
| DV867425 | 2 | 45787907 | 45787873 |  |  |
| DV867712 | 2 | 11494377 | 11493479 | 2 | Bd3 |
| DV867831 | 2 | 25508283 | 25508196 |  |  |
| DV867864 | 2 | 20792554 | 20792906 | 2 | Bd1, 4 |
| DV867908 | 2 | 52480863 | 52480946 |  |  |
| DV867939 | 2 | 7218301 | 7218453 |  |  |
| DV868191 | 2 | 21033688 | 21033740 |  |  |
| DV868386 | 2 | 34339496 | 34339419 |  |  |
| DV868415 | 2 | 49834388 | 49834740 |  |  |
| DV868462 | 2 | 42039673 | 42039736 |  |  |
| DV868512 | 2 | 46502209 | 46502009 |  |  |
| DV868569 | 2 | 15977039 | 15976552 | 2 | Bd1 |
| DV868618 | 2 | 52190423 | 52190047 |  |  |
| DV868699 | 2 | 18081321 | 18081370 |  |  |
| DV868717 | 2 | 58846361 | 58846404 |  |  |
| DV860413 | 3 | 57592315 | 57592604 |  |  |
| DV860467 | 3 | 41827196 | 41826633 |  |  |
| DV860484 | 3 | 14660906 | 14660670 |  |  |
| DV860500 | 3 | 26475457 | 26476433 |  | Bd1 |
| DV860566 | 3 | 43602264 | 43602433 |  |  |
| DV860613 | 3 | 9538980 | 9539361 |  |  |
| DV860681 | 3 | 55140095 | 55140373 |  |  |
| DV860756 | 3 | 24423176 | 24423053 |  |  |
| DV860895 | 3 | 55140137 | 55140373 |  |  |
| DV861045 | 3 | 55820244 | 55819898 |  |  |
| DV861113 | 3 | 36891442 | 36891133 |  |  |
| DV861170 | 3 | 5230478 | 5230712 |  |  |
| DV861212 | 3 | 28301395 | 28301269 |  |  |
| DV861458 | 3 | 49035386 | 49035420 |  |  |
| DV861464 | 3 | 35237938 | 35238426 |  |  |
| DV861493 | 3 | 45748962 | 45748911 |  |  |
| DV861509 | 3 | 20773241 | 20772793 |  |  |
| DV861526 | 3 | 4234010 | 4234423 |  |  |
| DV861537 | 3 | 53307319 | 53307496 |  |  |
| DV861784 | 3 | 5230712 | 5230469 |  |  |
| DV861786 | 3 | 6822935 | 6822678 |  |  |
| DV861842 | 3 | 33515698 | 33515503 |  |  |
| DV861859 | 3 | 18005976 | 18005900 |  |  |
| DV861876 | 3 | 54190217 | 54190548 |  |  |
| DV861883 | 3 | 3589294 | 3589356 |  |  |
| DV862048 | 3 | 44114069 | 44114417 |  | Bd1, 2, 4 |
| DV862087 | 3 | 51607353 | 51607297 |  |  |
| DV862175 | 3 | 52059922 | 52060115 |  |  |
| DV862209 | 3 | 27093561 | 27093458 |  | Bd4, 5 |
| DV862232 | 3 | 47189109 | 47189388 |  |  |
| DV862248 | 3 | 46634216 | 46634359 |  |  |
| DV862254 | 3 | 11310459 | 11309975 |  |  |
| DV862334 | 3 | 6584285 | 6584137 |  |  |
| DV862417 | 3 | 31678620 | 31678309 | 3 | Bd1, 2, 4, 5 |
| DV862562 | 3 | 53982648 | 53982619 |  |  |
| DV862591 | 3 | 34216918 | 34217129 |  |  |
| DV862624 | 3 | 2683106 | 2682823 |  |  |
| DV862689 | 3 | 41216693 | 41216912 |  |  |
| DV862703 | 3 | 54139041 | 54138920 |  |  |
| DV862767 | 3 | 2116200 | 2116120 |  |  |
| DV862948 | 3 | 29195056 | 29195153 |  |  |
| DV863002 | 3 | 53349136 | 53349260 |  |  |
| DV863101 | 3 | 54190304 | 54190548 |  |  |
| DV863192 | 3 | 54190241 | 54190546 |  |  |
| DV863431 | 3 | 36345148 | 36345300 |  |  |
| DV863446 | 3 | 13270142 | 13269770 |  |  |
| DV863555 | 3 | 55140258 | 55140373 |  |  |
| DV863594 | 3 | 37368021 | 37368100 |  |  |
| DV863664 | 3 | 53348929 | 53349260 |  |  |
| DV863673 | 3 | 49306759 | 49306504 |  |  |
| DV863873 | 3 | 17661430 | 17661745 |  |  |
| DV864207 | 3 | 14785676 | 14785599 |  |  |
| DV864229 | 3 | 58563247 | 58562887 |  |  |
| DV864230 | 3 | 11902479 | 11902181 |  |  |
| DV864327 | 3 | 2601565 | 2601748 |  |  |
| DV864343 | 3 | 337028 | 336766 |  |  |
| DV864382 | 3 | 49951702 | 49952106 |  |  |
| DV864458 | 3 | 58898957 | 58899115 |  |  |
| DV864525 | 3 | 46632856 | 46633079 |  |  |
| DV864540 | 3 | 21374341 | 21374373 |  |  |
| DV864553 | 3 | 14438066 | 14437928 |  |  |
| DV864557 | 3 | 47725037 | 47725109 |  |  |
| DV864561 | 3 | 41861655 | 41861108 | 3 |  |
| DV864592 | 3 | 9405379 | 9405482 | 9 | Bd2, 4, 5 |
| DV864600 | 3 | 52766934 | 52766564 |  |  |
| DV864630 | 3 | 24456747 | 24456584 |  |  |
| DV864680 | 3 | 1832061 | 1831933 |  |  |
| DV864793 | 3 | 56413098 | 56413248 |  |  |
| DV864818 | 3 | 50076027 | 50075975 |  |  |
| DV864850 | 3 | 52767894 | 52767791 |  |  |
| DV864994 | 3 | 44093484 | 44093418 |  |  |
| DV865022 | 3 | 41334364 | 41334141 |  |  |
| DV865074 | 3 | 15179324 | 15179068 |  |  |
| DV865130 | 3 | 55140018 | 55140373 |  |  |
| DV865247 | 3 | 6737570 | 6737609 |  |  |
| DV865446 | 3 | 3799323 | 3799259 |  |  |
| DV865545 | 3 | 52611933 | 52612227 |  |  |
| DV865674 | 3 | 28296881 | 28296365 |  | Bd3 |
| DV865689 | 3 | 38984160 | 38984084 |  |  |
| DV865701 | 3 | 59882148 | 59881463 |  | Bd1, 5 |
| DV865707 | 3 | 7587848 | 7588313 |  |  |
| DV865708 | 3 | 28296908 | 28296365 |  | Bd1 |
| DV865723 | 3 | 43626765 | 43626818 |  |  |
| DV865804 | 3 | 28296903 | 28296365 |  | Bd1 |
| DV865826 | 3 | 28296881 | 28296365 |  |  |
| DV865884 | 3 | 1260196 | 1260104 | 6 | Bd1, 2, 4 |
| DV866009 | 3 | 49035313 | 49035420 | 2 |  |
| DV866029 | 3 | 48566785 | 48566680 |  |  |
| DV866042 | 3 | 16249343 | 16249408 |  |  |
| DV866055 | 3 | 46867731 | 46867839 |  |  |
| DV866058 | 3 | 53308274 | 53308036 |  |  |
| DV866079 | 3 | 41216070 | 41216163 |  |  |
| DV866085 | 3 | 54190311 | 54190546 |  |  |
| DV866097 | 3 | 48293931 | 48293961 |  |  |
| DV866118 | 3 | 42077347 | 42077437 |  |  |
| DV866134 | 3 | 53986653 | 53986595 |  |  |
| DV866137 | 3 | 53212701 | 53212662 |  |  |
| DV866171 | 3 | 21962376 | 21962519 |  |  |
| DV866329 | 3 | 2116161 | 2116116 |  |  |
| DV866488 | 3 | 39621971 | 39621881 |  |  |
| DV866557 | 3 | 55686020 | 55686086 |  |  |
| DV866702 | 3 | 26473816 | 26473283 | 2 | Bd1, 5 |
| DV866764 | 3 | 36079096 | 36079123 |  |  |
| DV866879 | 3 | 55473254 | 55473386 |  |  |
| DV866906 | 3 | 50602678 | 50602552 |  |  |
| DV866945 | 3 | 5325172 | 5324854 |  |  |
| DV866947 | 3 | 19004342 | 19004259 |  |  |
| DV866994 | 3 | 45749061 | 45748911 |  |  |
| DV867088 | 3 | 27092988 | 27093144 | 2 | Bd4, 5 |
| DV867365 | 3 | 32196984 | 32196364 |  |  |
| DV867452 | 3 | 50072981 | 50073043 |  |  |
| DV867607 | 3 | 4791079 | 4790646 |  |  |
| DV867630 | 3 | 39010016 | 39009952 |  |  |
| DV867631 | 3 | 57693466 | 57693525 |  |  |
| DV867712 | 3 | 28306471 | 28307335 |  | Bd2 |
| DV867720 | 3 | 54698832 | 54698904 |  |  |
| DV867867 | 3 | 32000393 | 32000422 |  |  |
| DV867874 | 3 | 53307259 | 53307496 |  |  |
| DV867975 | 3 | 42609386 | 42609633 |  |  |
| DV868003 | 3 | 34849296 | 34849437 |  |  |
| DV868026 | 3 | 12516856 | 12516792 |  |  |
| DV868235 | 3 | 48294120 | 48294174 |  |  |
| DV868245 | 3 | 27092986 | 27093371 |  | Bd4, 5 |
| DV868314 | 3 | 995357 | 995185 |  |  |
| DV868382 | 3 | 34231467 | 34231259 |  |  |
| DV868445 | 3 | 53307889 | 53307984 |  |  |
| DV868526 | 3 | 38400870 | 38400966 |  |  |
| DV868603 | 3 | 44238425 | 44238525 |  | Bd4 |
| DV868608 | 3 | 35808972 | 35809225 |  |  |
| DV868655 | 3 | 36451259 | 36450614 |  |  |
| DV868696 | 3 | 33554205 | 33554502 |  | Bd1 |
| DV860562 | 4 | 5998061 | 5997972 |  |  |
| DV860573 | 4 | 12827393 | 12827931 |  | Bd1 |
| DV860617 | 4 | 23335470 | 23335842 |  |  |
| DV860816 | 4 | 6943097 | 6943307 |  |  |
| DV861225 | 4 | 2860959 | 2860524 |  |  |
| DV861247 | 4 | 41342398 | 41342428 |  |  |
| DV861347 | 4 | 9381465 | 9381570 |  |  |
| DV861636 | 4 | 4905440 | 4905332 |  |  |
| DV861734 | 4 | 7477414 | 7477380 |  |  |
| DV861753 | 4 | 32664644 | 32664666 |  |  |
| DV862048 | 4 | 42403184 | 42403529 |  | Bd1, 2, 3 |
| DV862209 | 4 | 7941422 | 7941479 |  | Bd3, 5 |
| DV862417 | 4 | 41377627 | 41377317 |  | Bd1, 2, 3, 5 |
| DV862668 | 4 | 3061596 | 3061518 |  |  |
| DV862928 | 4 | 35863629 | 35863411 |  |  |
| DV862938 | 4 | 40525915 | 40526007 |  |  |
| DV862973 | 4 | 3472143 | 3472495 | 16 | Bd2, 5 |
| DV863232 | 4 | 2311169 | 2311316 | 4 | Bd5 |
| DV863635 | 4 | 13767568 | 13767051 | 3 |  |
| DV864085 | 4 | 7547767 | 7547646 | 2 |  |
| DV864184 | 4 | 8423213 | 8423511 |  |  |
| DV864355 | 4 | 37802805 | 37802859 |  |  |
| DV864369 | 4 | 33709656 | 33709864 |  |  |
| DV864424 | 4 | 37613848 | 37613993 |  |  |
| DV864475 | 4 | 26439698 | 26440071 |  |  |
| DV864528 | 4 | 200144 | 199930 |  |  |
| DV864592 | 4 | 8131489 | 8131669 | 8 | Bd2, 3, 5 |
| DV864830 | 4 | 33887378 | 33887554 |  |  |
| DV864889 | 4 | 33709656 | 33709864 |  |  |
| DV864966 | 4 | 32725916 | 32725872 |  |  |
| DV865096 | 4 | 45035187 | 45035264 |  |  |
| DV865392 | 4 | 757131 | 757066 |  | Bd1 |
| DV865666 | 4 | 5569885 | 5570111 |  |  |
| DV865884 | 4 | 3951132 | 3951040 | 5 | Bd1, 2, 3 |
| DV865890 | 4 | 8422997 | 8423093 |  |  |
| DV865966 | 4 | 31049668 | 31049571 |  |  |
| DV866068 | 4 | 45035166 | 45035264 |  |  |
| DV866159 | 4 | 24618071 | 24617821 |  |  |
| DV866573 | 4 | 8423334 | 8423511 |  |  |
| DV866645 | 4 | 41721218 | 41721185 | 2 |  |
| DV866653 | 4 | 27015231 | 27015379 | 2 |  |
| DV866717 | 4 | 1071167 | 1071376 |  |  |
| DV866731 | 4 | 36215370 | 36215424 |  |  |
| DV866847 | 4 | 16568599 | 16568411 |  |  |
| DV866866 | 4 | 32295119 | 32294863 |  |  |
| DV866921 | 4 | 17458499 | 17458445 |  | Bd1 |
| DV866965 | 4 | 43773471 | 43773595 |  |  |
| DV867026 | 4 | 30355538 | 30355387 |  |  |
| DV867088 | 4 | 7942029 | 7941877 | 2 | Bd3, 5 |
| DV867116 | 4 | 37910205 | 37910075 |  |  |
| DV867418 | 4 | 3357310 | 3357338 |  |  |
| DV867542 | 4 | 36215197 | 36215424 |  |  |
| DV867756 | 4 | 35744568 | 35744514 |  |  |
| DV867864 | 4 | 3420284 | 3420648 |  | Bd1, 2 |
| DV867978 | 4 | 41146228 | 41146272 |  |  |
| DV868017 | 4 | 29882056 | 29881815 | 2 |  |
| DV868027 | 4 | 30354683 | 30355233 |  |  |
| DV868078 | 4 | 41808838 | 41808767 |  |  |
| DV868245 | 4 | 7546855 | 7547238 |  | Bd3, 5 |
| DV868253 | 4 | 7497078 | 7496803 |  |  |
| DV868381 | 4 | 8423364 | 8423464 |  |  |
| DV868411 | 4 | 8423333 | 8423512 |  |  |
| DV868417 | 4 | 23699443 | 23699364 |  |  |
| DV868603 | 4 | 43417370 | 43417462 |  | Bd3 |
| DV868616 | 4 | 39121245 | 39121177 |  |  |
| DV868639 | 4 | 17457309 | 17457234 |  |  |
| DV860321 | 5 | 556999 | 556867 |  |  |
| DV862105 | 5 | 26152340 | 26152471 |  |  |
| DV862209 | 5 | 5280265 | 5280384 |  | Bd3, 4 |
| DV862417 | 5 | 2371119 | 2371408 |  | Bd1, 2, 3, 4 |
| DV862454 | 5 | 9879775 | 9879416 |  |  |
| DV862599 | 5 | 13475166 | 13475477 |  |  |
| DV862716 | 5 | 22418989 | 22419077 |  |  |
| DV862906 | 5 | 9617332 | 9617129 |  |  |
| DV862933 | 5 | 22419478 | 22419344 |  |  |
| DV862973 | 5 | 323610 | 323650 | 6 | Bd2, 4 |
| DV863161 | 5 | 18173955 | 18173595 |  | Bd1 |
| DV863232 | 5 | 1636986 | 1637171 | 5 | Bd4 |
| DV863372 | 5 | 20009427 | 20009345 |  |  |
| DV863522 | 5 | 15228513 | 15228449 |  |  |
| DV863707 | 5 | 25383635 | 25383680 |  |  |
| DV863732 | 5 | 26268894 | 26268812 |  |  |
| DV863777 | 5 | 17060599 | 17060864 |  |  |
| DV863851 | 5 | 19043205 | 19042880 |  |  |
| DV863880 | 5 | 9812091 | 9811993 |  |  |
| DV863987 | 5 | 19757998 | 19758208 |  |  |
| DV864085 | 5 | 5280260 | 5280384 |  |  |
| DV864145 | 5 | 9810291 | 9810267 |  |  |
| DV864301 | 5 | 6768918 | 6768952 |  |  |
| DV864385 | 5 | 3127656 | 3127514 |  |  |
| DV864567 | 5 | 9618288 | 9618248 |  |  |
| DV864592 | 5 | 2865773 | 2865950 | 5 | Bd2, 3, 4 |
| DV864607 | 5 | 17666190 | 17666155 |  |  |
| DV864930 | 5 | 17418268 | 17418535 |  |  |
| DV865067 | 5 | 23463062 | 23462980 |  | Bd2 |
| DV865147 | 5 | 21116726 | 21116851 |  |  |
| DV865243 | 5 | 19226028 | 19225990 |  |  |
| DV865579 | 5 | 22418954 | 22419077 |  |  |
| DV865701 | 5 | 5536 | 4659 | 30 | Bd1, 3 |
| DV865833 | 5 | 19197894 | 19197970 |  |  |
| DV865909 | 5 | 16097438 | 16097918 |  |  |
| DV866106 | 5 | 9704864 | 9704790 |  |  |
| DV866389 | 5 | 20009378 | 20009345 |  |  |
| DV866651 | 5 | 14833840 | 14833799 |  |  |
| DV866689 | 5 | 12974472 | 12974588 |  |  |
| DV866702 | 5 | 25646469 | 25647003 |  | Bd1, 3 |
| DV867088 | 5 | 5281337 | 5281181 |  | Bd3, 4 |
| DV867307 | 5 | 22015019 | 22015084 |  |  |
| DV867384 | 5 | 4345446 | 4345302 |  |  |
| DV867516 | 5 | 16032678 | 16032634 |  |  |
| DV867620 | 5 | 15704364 | 15704614 |  |  |
| DV867663 | 5 | 10410936 | 10411180 |  |  |
| DV868245 | 5 | 5281340 | 5280955 |  | Bd3, 4 |
| DV868429 | 5 | 22094662 | 22094785 |  |  |
| DV868456 | 5 | 25681656 | 25681962 |  |  |

^a^ *B.* *distachyon* chromosome where the creeping bentgrass EST was found as ortholog.

^b^ Location of the alignment between the marker and the *B.* *distachyon* chromosome (start and end correspond to base pair number).

^c^ Number of multiple matches of creeping bentgrass EST orthologs within one *B.* *distachyon* chromosome.

^d^ Additional *B.* *distachyon* chromosome where the creeping bentgrass EST was found as ortholog.
